# The Computational Bottleneck of Basal Ganglia Output (and What to Do About it)

**DOI:** 10.1523/ENEURO.0431-23.2024

**Published:** 2025-04-18

**Authors:** Mark D. Humphries

**Affiliations:** School of Psychology, University of Nottingham, Nottingham, UK

## Abstract

What the basal ganglia do is an oft-asked question; answers range from the selection of actions to the specification of movement to the estimation of time. Here, I argue that *how* the basal ganglia do what they do is a less-asked but equally important question. I show that the output regions of the basal ganglia create a stringent computational bottleneck, both structurally, because they have far fewer neurons than do their target regions, and dynamically, because of their tonic, inhibitory output. My proposed solution to this bottleneck is that the activity of an output neuron is setting the weight of a basis function, a function defined by that neuron’s synaptic contacts. I illustrate how this may work in practice, allowing basal ganglia output to shift cortical dynamics and control eye movements via the superior colliculus. This solution can account for troubling issues in our understanding of the basal ganglia: why we see output neurons increasing their activity during behavior, rather than only decreasing as predicted by theories based on disinhibition, and why the output of the basal ganglia seems to have so many codes squashed into such a tiny region of the brain.

## Significance Statement

The basal ganglia are implicated in functions ranging from action selection to timing and in dysfunctions from Parkinson’s disease to obsessive compulsive disorder. Yet, however, the basal ganglia cause these functions and dysfunctions they must do so through a group of neurons that are dwarfed in number by both their inputs and their output targets. Here, I lay out this bottleneck problem for basal ganglia computation, and propose a solution to how their outputs can control their many targets. That solution rethinks the output connections of the basal ganglia as a set of basis functions. In doing so, it provides explanations for previously troubling data on basal ganglia output, and strong predictions for how that output controls its targets. 

## Introduction

How do the basal ganglia do any useful work? I will argue here that they suffer from a severe computational bottleneck. Their output nuclei, through which they connect with the rest of the brain, are markedly smaller than both their input sources and output targets. Moreover, the standard view is that the basal ganglia’s output nuclei encode by disinhibition, by the cessation of their inhibitory output (Chevalier et al., 1985; Chevalier and Deniau, 1990; Hikosaka et al., 2000; Basso and Sommer, 2011), which provides limited capacity for carrying information. Yet the moment-to-moment dynamics of the basal ganglia are implicated in a lengthy list of proposed functions, including action selection (Redgrave et al., 1999; Klaus et al., 2019), motor program selection (Mink, 1996), kinematic gain control (Turner and Desmurget, 2010; Park et al., 2020), perceptual decision making (Ding and Gold, 2013; Yartsev et al., 2018), duration estimation (Buhusi and Meck, 2005; Gouvêa et al., 2015; Mello et al., 2015; Monteiro et al., 2023), signal routing (Stocco et al., 2010), and more. How one or more such complex functions are enacted through an output signal that has limited capacity in both size and dynamics is unclear.

I will begin here by defining this computational bottleneck problem, first detailing the anatomical expansion between the basal ganglia output nuclei and their targets, then arguing that the disinhibition view of basal ganglia output is limited. This sets up two fundamental problems for the basal ganglia output: one, how does it re-expand? And, two, what dynamics does it use to code?

I propose a solution to both these size and coding problems: that the basal ganglia output neurons’ projections to their targets are a set of basis functions, and the output neurons’ activity sets the weights of those functions. All of these ideas will be elaborated below. This solution explains both how basal ganglia output can expand to the same scale as its targets, and why it would need to both decrease and increase its activity. It can also account for troubling features of basal ganglia output, including why it has so many apparently different coding schemes. Consequently, it is a step towards reconciling the basal ganglia’s many apparent functional roles and may shed further light on why dysfunction of the basal ganglia is implicated in so many neural disorders.

## The computational bottleneck problem

### The structural bottleneck

In rodents, the basal ganglia output nuclei are traditionally considered to be the subtantia nigra pars reticulata (SNr) and the entopeduncular nucleus (EP). In primates, the latter is equivalent to the internal segment of the globus pallidus (GPi). Regardless of their names, these all share common anatomical properties: they receive input from the striatum and output to structures including multiple regions of the thalamus, the superior colliculus, and the upper brainstem (Deniau and Chevalier, 1992; McElvain et al., 2021).

The striatum dwarfs the output nuclei. In rats, the striatum in one hemisphere contains around 2.8 million neurons, whereas the SNr and EP combined contain around 30,000 (Oorschot, 1996), smaller by a factor of 100. In mice, the striatum contains about 400,000 projection neurons and the SNr around 12,000 neurons (numbers from the Blue Brain Project Cell Atlas, Rodarie et al., 2022); assuming that half of all projection neurons are D1-expressing and so project to the SNr, this gives a ratio of about 16:1 striatal projection neurons projecting to every SNr neuron in mice. This convergence of striatal projection neurons onto the basal ganglia output nuclei is well known, but we know little about the sizes of the target regions of the output nuclei.

To better understand the scale of the structural bottleneck, I used data from the Allen Mouse Brain Connectivity Atlas (Oh et al., 2014) to first identify a complete set of projection targets of the mouse SNr. The Atlas contains six experiments in which a fluorescent anterograde tracer was injected into the right SNr and filled at least 20% of its volume. For each experiment, I found which of a set of 295 nonoverlapping target brain regions had evidence of projections from the SNr, by checking if the density of tracer in that region exceeded some threshold. A threshold was necessary to eliminate image noise and other artefacts: without one, all 295 regions contained fluorescent pixels, implausibly implying the SNr projects to every area of gray matter in the mouse brain, from medulla to olfactory bulb (Methods). The number of neurons in each retained target region was found from the Blue Brain Project’s Cell Atlas for the mouse brain (Rodarie et al., 2022).

**Figure 1.**
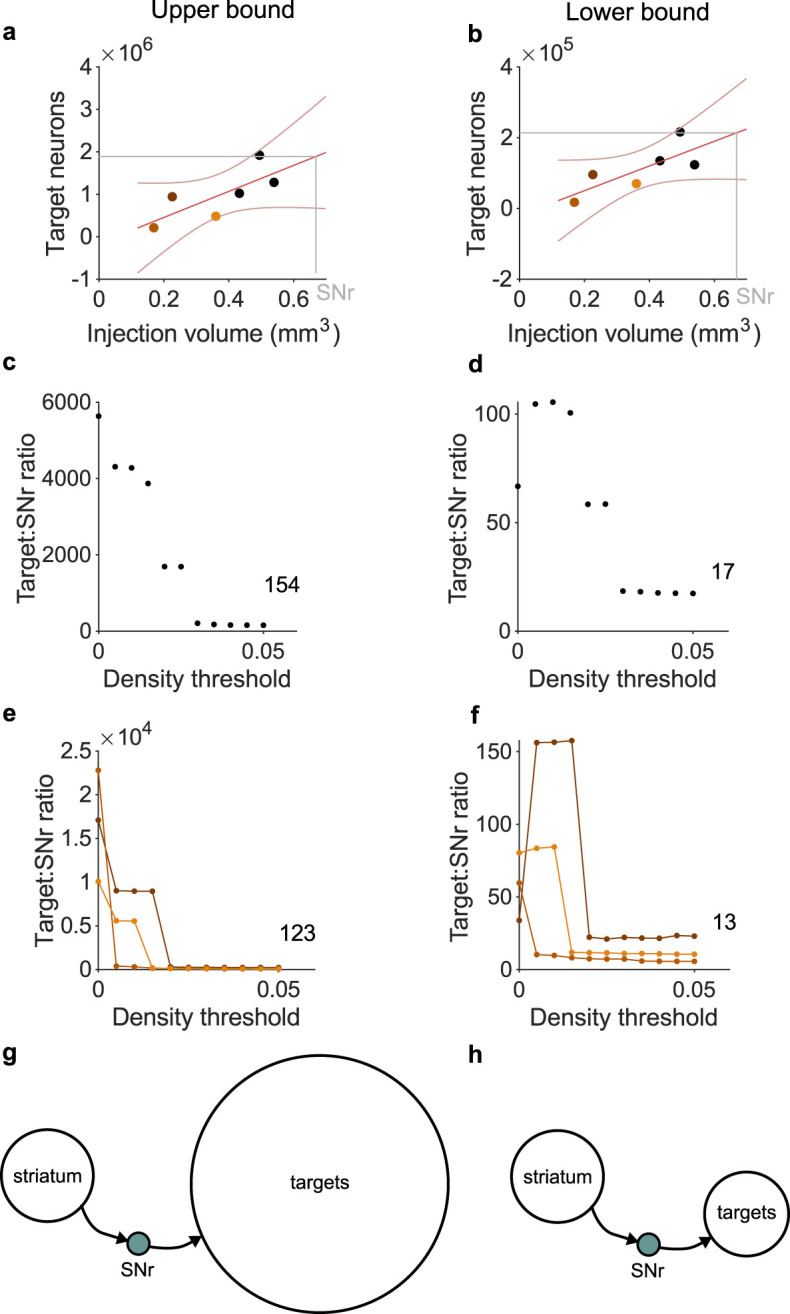
The structural bottleneck of basal ganglia output in the mouse. ***a***, The total number of neurons in SNr target regions scales with the volume of tracer injection. Each symbol is an estimate from one tracing experiment of the Allen Mouse Brain Atlas; non-black symbols are experiments with more than 90% of the injection within SNr. Red lines show linear fit and 95% confidence interval. Gray line is the extrapolated total neurons in SNr targets from its volume. ***b***, As panel a, but estimating the targeted neurons in each region from the density of tracer in that region’s volume (Methods). ***c***, The ratio of target neurons to SNr neurons for a range of thresholds on the minimum tracer density needed to include a target region. Data in panel a is for a threshold of 0.05. Number is the asymptotic estimate of the ratio. ***d***, As panel c, but estimating the targeted neurons in each region from the density of tracer in that region’s volume (Methods). ***e***, As panel c, for each of the three tracer experiments with injection volume confined to the SNr (non-black symbols in panel a). Here, the expected number of neurons in SNr target regions is computed by scaling that experiment’s total number of target neurons by the proportion of SNr filled by the injection. Number is the asymptotic estimate of the ratio averaged over the three experiments. ***f***, As panel e, but estimating the targeted neurons in each region from the density of tracer in that region’s volume. ***g***, Schematic of the upper bound of the SNr bottleneck. Target size from panel e; striatal D1R population estimated at 200,000 neurons (see text). ***h***, As for panel g, for the lower bound of the SNr bottleneck, target size from panel f.

The total number of neurons in SNr target regions scaled with the size of the tracer’s injection volume (Fig. 1*a*). All injection volumes were smaller than the volume of the mouse’s SNr. Fitting a linear model to the scaling let me extrapolate to the number of neurons targeted by the whole SNr (Fig. 1*a*, gray lines), and so estimate the ratio of target neurons to SNr neurons. This ratio fell to a stable value with increasingly stringent thresholds for eliminating noise (Fig. 1*c*), estimated as 154:1.

The extrapolation to the whole SNr’s projection was based on three experiments that had less than 40% of their injection volume inside the SNr (black symbols in Fig. 1*a*), potentially including neighboring regions of the SNr that have different connection patterns. Weighting the linear model fit by the proportion of the injection inside the SNr gave practically identical ratios of target to SNr neurons (not shown). Estimating the number of target neurons directly for each of the three tracer injections almost wholly within the SNr (non-black symbols in Fig. 1*a*), by extrapolating from the volume of their injection (Methods), also resulted in similar and stable ratios for the total number of target neurons to the number of SNr neurons (Fig. 1*e*).

The total number of neurons in the target regions is an upper bound on the number of connections made by SNr neurons. To estimate a lower bound, I approximated the arborization of the axons from SNr in the target region by the volume density of the tracer in that region, scaling the number of neurons in each target region by the proportion of its volume occupied by the tracer (Methods). This lower bound estimate of target neurons also scaled with the tracer’s injection volume (Fig. 1*b*), reached a stable value with increasing noise threshold (Fig. 1*e*), and was robust to alternative calculation using the three within-SNr experiments (Fig. 1*f*).

The estimated expansion from the basal ganglia output nucleus SNr to its targets ranges from about 1:154 down to 1:13 (Fig. 1). Even the lower bound on this expansion is thus about as large as the compression of inputs from striatum: the basal ganglia’s output is then a considerable bottleneck, compressing its inputs by at least 10:1, and re-expanding them in its output targets by at least 1:10 (Fig. 1*g,h*).

### The dynamic bottleneck (or, why not disinhibition)

Basal ganglia output neurons are constantly active. In rodents, they typically average 30 spikes/s; in primates, around 60 spikes/s. They are also all GABAergic. This constant stream of high-frequency GABA release on to their target neurons has naturally led to the assumption that they constantly inhibit their targets. From that has followed the disinhibition hypothesis (Chevalier et al., 1985; Deniau and Chevalier, 1985; Chevalier and Deniau, 1990) that releasing this inhibition is key to how the basal ganglia encode information, by allowing their target neurons to respond to their inputs. This signaling by disinhibition is the basis for most prominent conceptual (Mink, 1996; Redgrave et al., 1999; Hikosaka et al., 2000) and computational (Gurney et al., 2001; Frank, 2005; Humphries et al., 2006; Leblois et al., 2006; Bogacz and Gurney, 2007; Vitay and Hamker, 2010; Liénard and Girard, 2014; Lindahl and Hellgren Kotaleski, 2016; Dunovan et al., 2019) models of the basal ganglia.

Yet, it is a myopic view of basal ganglia output that places a strong limitation on the potential dynamic range of the output neurons, allowing coding by only a decrease in activity, and then often reduced to just whether the activity is on or off, a binary signal (e.g., Hikosaka et al., 2000). There is no role for increases in activity, or changes to the patterns of activity (there are other theories: for example, there is evidence that the output of the basal ganglia in songbirds controls the timing of activity in their thalamic targets Goldberg et al., 2013). This compounds the structural bottleneck by then further limiting how each neuron can send information. For example, taking the binary on/off signal literally, disinhibition reduces the information coding capacity of the whole basal ganglia output to just one bit per neuron, a few thousand bits in total.

## A solution: basal ganglia outputs are dynamic weights

The basal ganglia’s computational bottleneck problem is, then, that we have a limited number of free parameters—the output neurons and their dynamical range—compared to the number of outputs that we need to control. My proposed solution to this problem is a reframing of what the basal ganglia output encodes.

I propose that the basal ganglia’s output connections are best understood as basis functions, and the level of basal ganglia output activity sets the weights on those functions. Let’s unpack those ideas, starting with a definition of basis functions.

**Figure 2.**
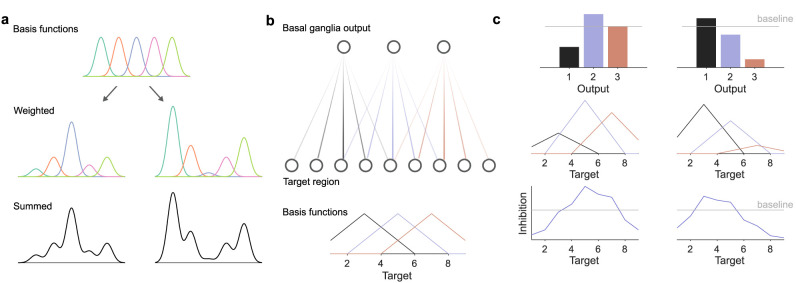
Basis functions and basal ganglia output. ***a***, Schematic of basis functions. A range of values is tiled by a set of basis functions, here five Gaussians (top). Each basis function’s contribution is controlled by a single weight: the middle panels show two different weightings. Summing these weighted basis functions creates a continuous function spanning the range of values (bottom), controlled by just five parameters, the weights on the basis functions. ***b***, Basal ganglia output connections define basis functions. Top: idealized network showing distribution of basis function strengths, fanning from the output nuclei to a larger target region; colour intensity is proportional to strength. Bottom: the basis functions created by the connection strengths. ***c***, Basal ganglia output activity parameterizes the basis functions. Using the network in panel b, two examples of how basal ganglia output activity (top) scales each neuron’s basis function (middle), which when summed as the input to each target creates a continuous inhibition function (bottom). Gray line indicates baseline output activity (top) and consequent inhibition of targets (bottom).

Basis functions are a typical solution for how to use a few parameters to control a large range of output. Figure 2*a* shows the key ideas. We first tile the output range we want to control with a set of basis functions, such as the five Gaussians in the example of Figure 2*a*. Each basis function has a single weight that sets its contribution, such as the amplitude of a Gaussian (Fig. 2*a*, middle). Summing basis functions of different weights can then create many different output functions over a large range of outputs (Fig. 2*a*, bottom). Basis functions thus create an expansion from a few controllable parameters—the weights—to a much larger target space.

Now consider that the connections of the basal ganglia output neurons will have a distribution of strengths (Fig. 2*b*, top), the strength of a connection between an output neuron and its target being the product of the number of synapses and the conductances of those synapses. The idea then is that this distribution of strengths defines the basis functions (Fig. 2*b*, bottom).

Consequently, the amplitude of basal ganglia output activity sets the weights of those basis functions. Figure 2*c* shows two examples of what this would look like: a particular vector of basal ganglia output activity scales the basis functions created by the output strengths; when summed at each target, the larger target region as a whole receives a continuous function of inhibition, specified by far fewer basal ganglia output neurons than target neurons.

This idea rests on just two assumptions: that connections of the output neurons have a distribution of strengths, and that these distributions overlap. It seems vanishingly unlikely that a given output neuron has an identical effective influence on each of its target neurons, so a distribution of strengths seems reasonable. And because the output of the basal ganglia is topographically organized (Deniau and Chevalier, 1992; Hoover and Strick, 1993; Lee et al., 2020; Foster et al., 2021; McElvain et al., 2021), with adjacent neurons projecting to adjacent targets, we might also reasonably expect these distributions of strengths to overlap. Beyond that, I emphasize that the schematics in Figure 2 are for illustration, not theory: I’m not claiming that distributions of output strengths have to be symmetric, nor that their “centers” are distributed equidistant from each other in some topographic space, nor that the distributions have to be the same, nor that they have to follow any specific basis function used in the literature (such as radial basis functions). Rather, the theory proposed is perhaps best expressed as: basal ganglia output activity is a dynamic weight on some function defined by the strengths of the output connections. Let’s now state the most general form of the theory and derive some general predictions from it.

## The general form of the theory and its predictions

Consider that *b* basal ganglia output neurons project to a set of *n* target neurons. We have already established that *b* < *n*, the structural bottleneck. The theory proposes that the goal of basal ganglia output is to create a specific function of inhibition across those *n* target neurons, which we can describe in a *n*-dimensional vector **f** with entries *f*_*i*_ ≤ 0. The theory can thus be expressed as the linear system

**Figure 3.**
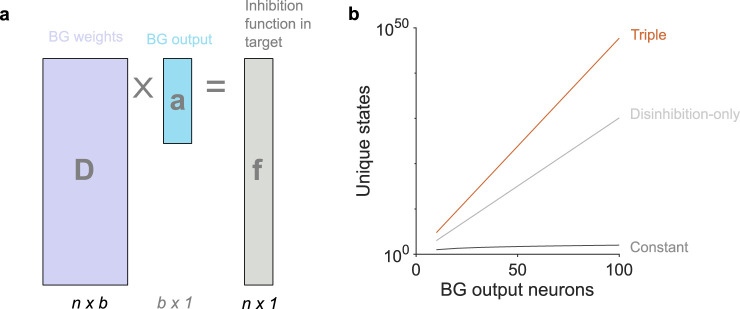
Linear system model of basal ganglia output. ***a***, Schematic of the linear model in Equation 1. ***b***, Scaling of the number of possible functions **f** defined by basal ganglia outputs. Each line give the number of unique functions possible with that number *b* of output neurons. Different lines correspond to the different number of states each output neuron can meaningfully be in: constant (1); disinhibition (2: on/off); triple (3^*b*^) is up/down/unchanged.

Da=f,

where **a** is the *b*-dimensional vector of basal ganglia output activity, and **D** is the *n* × *b* matrix of connection weights from the basal ganglia output to the set of target neurons (Fig. 3*a*). Their values are also constrained: *a*_*i*_ ≥ 0 as neural activity cannot be negative, and *D*_*ij*_ ≤ 0 because basal ganglia output is inhibitory. Matrix **D** defines the basis functions, one column per output neuron. For example, in the schematic model of Figure 2*b* each column of **D** is a shifted version of the same, symmetric basis function. Thus the vector **a** of basal ganglia activity are the dynamic weights on the basis functions **D** that gives the target function **f**.

### Prediction of non-uniform inhibition from uniform output

Basal ganglia output neurons **a** are tonically active, firing at rest at a rate of around 30 spikes/s in rodents and 60 spikes/s in primates. That **a** has non-zero values means a non-trivial target function **f** is always defined.

However, many theories implicitly assume that this tonic firing necessarily means there is a uniform level of inhibition, such that all values of **f** are the same. This is implied by theories that tonic inhibition defines the “no-go” or “off” signal for selecting responses, actions, or motor programs (Mink, 1996; Redgrave et al., 1999; Hikosaka et al., 2000). The model in Equation 1 shows this is only true if the rows of **D** have the same sum. But this is unlikely as only the columns of **D**, being the projections of each output neuron, are defined by development and plasticity. Consequently, the model predicts that tonic activity of a set of output neurons causes a nonuniform inhibition of their targets.

### Prediction of increased output activity

Traditionally, it is the cessation of this tonic inhibition, the disinhibition, that has been the basis for key conceptual (Mink, 1996; Redgrave et al., 1999; Hikosaka et al., 2000) and computational (Gurney et al., 2001; Frank, 2005; Humphries et al., 2006; Leblois et al., 2006; Bogacz and Gurney, 2007; Vitay and Hamker, 2010; Liénard and Girard, 2014; Lindahl and Hellgren Kotaleski, 2016; Dunovan et al., 2019) models of the basal ganglia’s function.

The model offered in Equation 1 places no constraints on the values of **a** around the baseline tonic activity. Rather, the tonic activity values of **a** define a default **f** from which behaviorally necessary changes to **f** must occur. This predicts that both increases as well as decreases in output neuron activity can change **f** when basal ganglia output must cause or influence some behavioral event.

This prediction is borne out by data. Basal ganglia output neuron activity does increase in many tasks (Gulley et al., 1999; Handel and Glimcher, 1999; Gulley et al., 2002; Sato and Hikosaka, 2002; Jin and Costa, 2010; Fan et al., 2012; Rossi et al., 2016; Schwab et al., 2020) and the increases are as equally time-locked to action as the decreases (Sato and Hikosaka, 2002; Jin and Costa, 2010; Fan et al., 2012). In some reports output neuron activity can seemingly continuously encode parameters both above and below the nominal “tonic” firing rate (Barter et al., 2015).

Allowing for increased output activity increases the basal ganglia’s scope for control. Restricting ourselves to classic disinhibition allows just two output states, on and off. The number of possible unique output combinations is then 2^*b*^ (Fig. 3*b*). But adding just one more output state, the increase above the tonic level, makes the number of unique input combinations 3^*b*^ (Fig. 3*b*): at 100 output neurons this triple state can achieve more than 5 × 10^47^ unique dynamic weight combinations and hence that many different functions of inhibition **f**. Consequently, even a small group of basal ganglia output neurons could control a wide repertoire of states in its target structures.

### Prediction of low variability in output activity

Stating the theory as the linear system Equation 1 lets us ask an interesting question: is basal ganglia output degenerate? That is, in some behavioral event for which the basal ganglia are necessary, can different combinations of increases and decreases of output neuron activity achieve the same behavioral effect?

Let’s assume that the same behavioral effect means achieving the same target function **f**. Then we are asking how many solutions exist to Equation 1 (Druckmann and Chklovskii, 2012): how many different basal ganglia outputs **a** achieve the same target function **f**.

A heterogeneous linear system like Equation 1 can have no, one, or an infinite number of such solutions. As we are interested in events where the basal ganglia have a necessary role, then by definition we are interested in the set of **f** that can be achieved by the basal ganglia output given **D**. So there must be at least one solution **a** for a given, behaviorally relevant, target function **f**. But is there more than one?

It seems unlikely. This is because the matrix of connection weights **D** is almost certainly full rank, having no linearly dependent columns. For structured basis functions, where each column of **D** is approximately a shifted version of the same function (like Fig. 2*a*), we can guarantee that **D** is full rank by construction (Methods). For random basis functions, selecting the values of **D** from a wide range of symmetric probability distributions would guarantee it was full rank (Rudelson and Vershynin, 2009). A linear system with full rank **D** has at most one solution. Thus, either the basal ganglia output connections have a genetically defined low-rank structure or there is only one basal ganglia output **a** that can achieve a given target function of inhibition **f**.

Having exactly one solution **a** to Equation 1 predicts that individual basal ganglia neurons would show little variability between repeated behavioral events that need the same **f**. I am proposing here that the necessary solution to **a** is for each behavioral event, so the predicted time-scale of this variation is around the gross changes in firing rate time-locked to an event, not precise spike-timing.

Conversely, observing considerable variability in basal ganglia output activity between repeated events would imply either that **f** is not the goal of basal ganglia output, so the theory here is incorrect, or that the connections of the basal ganglia to their targets are linearly dependent and so basal ganglia output is redundant. This in turn would imply strong constraints on how basal ganglia output is wired, in order to achieve this linear dependence.

I am unaware of convincing data either way. While there is a considerable literature on single neuron activity in both rodent SNr (e.g., Gulley et al., 1999, 2002; Bryden et al., 2011; Fan et al., 2012) and primate GPi/SNr (e.g., Handel and Glimcher, 1999; Sato and Hikosaka, 2002; Nevet et al., 2007; Sheth et al., 2011) during tasks, I am not aware of any that have quantified the trial-to-trial variability in that activity during exact repetition of a behavior for which the basal ganglia are necessary. Close examination of example raster plots of individual SNr neurons aligned to the onset of eye movements (e.g., Handel and Glimcher, 1999; Sato and Hikosaka, 2002) suggests that gross changes of activity are highly consistent between trials, unlike, say, the rate variation of individual cortical neurons between repeated sensory stimuli (Tolhurst et al., 1983).

## How the solution could work in practice

Let’s illustrate the idea of basal ganglia outputs as dynamic weights in two concrete instantiations: the control of cortical state by basal ganglia output to thalamus; and the control of superior colliculus’ coding of saccade target by its inputs from the SNr.

### Basal ganglia output control of the repertoire of cortical states

The basal ganglia’s output to the thalamus is the main focus of much theorizing because of its potential to control the dynamics of the cortical targets of those thalamic regions (e.g., Humphries and Gurney, 2002; Frank, 2005; Dunovan et al., 2019; Möller and Bogacz, 2019; Athalye et al., 2020; Logiaco et al., 2021). But the thalamic regions contain more neurons than the basal ganglia output nuclei, and the thalamus in turn is dwarfed by the numbers of cortical neurons (consider, for example, that of all the synapses arriving onto layer 4 neurons in the visual cortex, thalamic synapses make up just 5% of the total Peters and Payne, 1993). Here I illustrate how the idea of dynamic weights defined by the combinations of a few basal ganglia output neurons can allow control of cortical dynamics. In general, the problem of how a few inputs can drives the states of a larger dynamical system is studied under the control theory concept of *controllability* (Sontag and Sussmann, 1997; Liu et al., 2011; Gu et al., 2015; Kao and Hennequin, 2019); an interesting extension of the work here would use controllability approaches to identify what form of target function **f** is ultimately necessary to control cortical states, and which elements of the cortical circuit must be targeted to do so.

**Figure 4.**
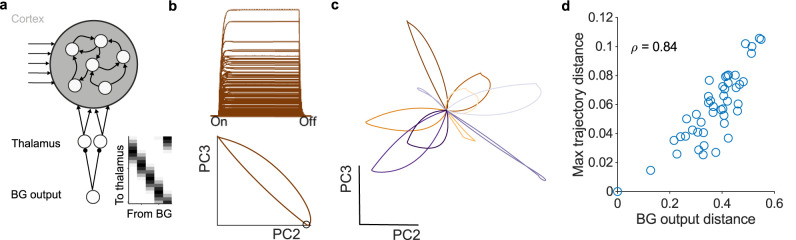
Basal ganglia output control of cortical dynamics. ***a***, Schematic of the recurrent neural network (RNN) model, its inputs from other cortical regions (arrows) and its inputs from thalamus. Basal ganglia output to thalamus is a set of overlapping symmetric basis functions (inset; gray-scale indicates strength of connection, white indicates no connection). Thalamus projects to 10% of the cortical RNN units. Example simulations use 5 basal ganglia outputs, 20 thalamic units, and 200 cortical units. ***b***, Example response of all RNN units to stepped input (top), and projection of that RNN activity into a low-dimensional space (PC: principal component). The trajectory of low-dimensional activity captures the move away from and return to baseline activity (black dot). ***c***, Trajectories of RNN activity in response to different basal ganglia outputs. Each line plots the trajectory in response to a different basal ganglia output vector, with all other inputs held constant. Output vectors were sampled from a uniform distribution centered on tonic activity, modelling both increases and decreases of output. ***d***, Variation in basal ganglia output maps to variation in the trajectories of RNN activity. *ρ*: Spearman’s rank.

Let’s consider a recurrent neural network (RNN) to model a region of cortex, as these nicely capture the basic problem: a network of mixed excitatory and inhibitory neurons that is capable of producing complex dynamics (Fig. 4*a*). A brief step in input to this network produces a population response (Fig. 4*b*, top). We can characterize this population response by the trajectory it creates in a low-dimensional space (Fig. 4*b*, bottom). Such trajectories of neural activity in cortex correspond to specific arm movements (Churchland et al., 2012; Gallego et al., 2017; Rodriguez et al., 2024), elapsed durations (Voitov and Mrsic-Flogel, 2022), or choices (Harvey et al., 2012).

A small fraction of these inputs, 10%, are from the thalamus. These thalamic inputs are in turn controlled by a small handful of basal ganglia output neurons, whose outputs create a set of basis functions to control thalamic activity. These basis functions are symmetric, overlapping, and tiled in a ring as shown in Figure 4*a* (inset). The Appendix discusses the constraints on the number of thalamic neurons and basal ganglia weight distributions implied by this model.

Despite the small number of basal ganglia outputs they are sufficient to qualitatively change the state of the cortical circuit. With all other inputs held the same, different vectors of basal ganglia output create different trajectories of activity in cortex (Fig. 4*c*). They do so by creating different functions of inhibition in the thalamus, defined by both decreases (disinhibition) and increases in basal ganglia output activity. Crucially, we see that these changes in trajectory would alter cortical coding (of an arm movement, a delay, a choice) without need for that coding in the basal ganglia output.

Work in songbirds has argued that basal ganglia activity is necessary to explore the repertoire of potential movements, including the variation of syllable generation in songs (Ölveczky et al., 2005; Singh Alvarado et al., 2021). The dynamic weights view is also consistent with this argument: the trajectories of activity in the cortical circuit vary in proportion to the distance between the output vectors of the basal ganglia (Fig. 4*d*). So greater variation in basal ganglia output could map to greater exploration of the cortical repertoire of dynamics, and thus to motor behavior or choices (de A Marcelino et al., 2023).

### How output neurons can control superior collicular activity to influence the orientation of the eyes and body

Another major target of basal ganglia output is the intermediate layers of the superior colliculus. This structure plays a key role in orienting the eyes and body (Hikosaka et al., 2000; Felsen and Mainen, 2008; Villalobos and Basso, 2022), with activity in its intermediate layer acting as a command signal for eye movements to a particular location (Hikosaka et al., 2000). Much ink has thus been spilt on how the inhibitory signals emanating from the basal ganglia to the superior colliculus may in turn control the direction of gaze (Hikosaka and Wurtz, 1985; Jiang et al., 2003; Girard and Berthoz, 2005; Basso and Sommer, 2011).

Most theories agree on the following (Hikosaka et al., 2000; Jiang et al., 2003; Girard and Berthoz, 2005; Chambers et al., 2011; Thurat et al., 2015). The intermediate, or motor, layer of the superior colliculus represent the direction of gaze in two-dimensional retinotopic co-ordinates. For convenience we’ll consider them as a Cartesian grid of (x,y) positions in a two-dimensional plane. Neural activity in the intermediate layer thus represents a motor command to direct gaze towards the location represented by the active neurons (Anderson et al., 1998; Lee et al., 1988). Basal ganglia inhibition of these collicular neurons is able to suppress changes in gaze direction to remembered locations (Mahamed et al., 2014) and possibly stimulus-driven locations. Consequently, a pause in basal ganglia output directed at collicular neurons representing a particular location allows the change in gaze to happen (Hikosaka and Wurtz, 1985).

**Figure 5.**
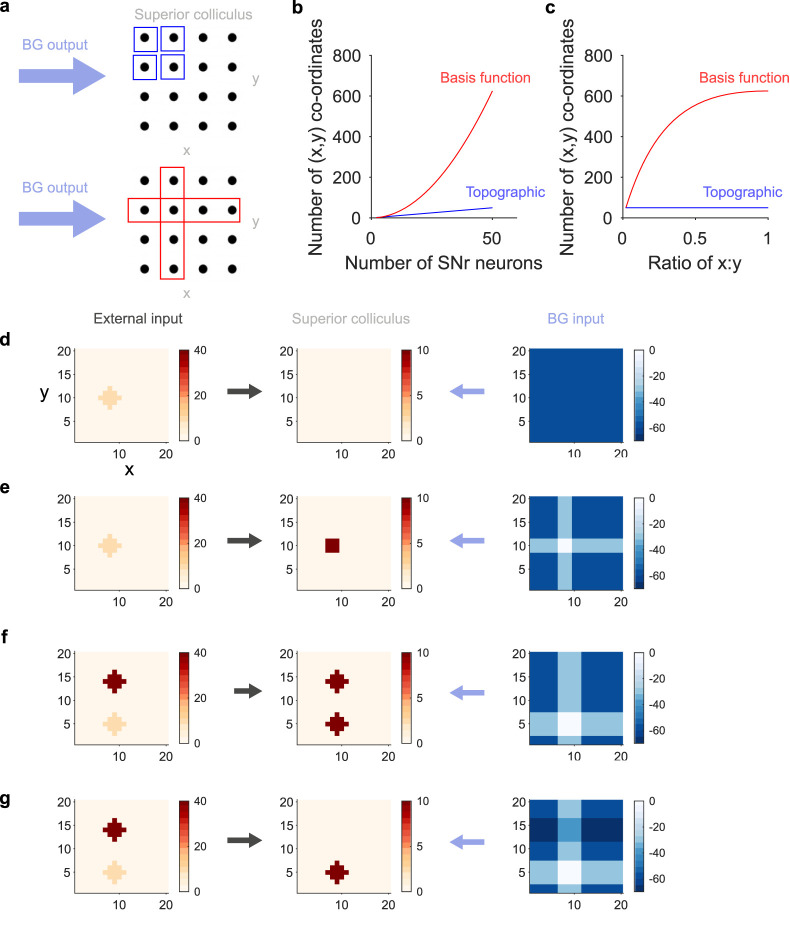
Basal ganglia output control of superior colliculus. ***a***, Potential schemes for basal ganglia inhibition of saccade targets in the intermediate layers of superior colliculus. The black circles are collicular neurons representing saccade targets in Cartesian co-ordinates. Top: topographic mapping of basal ganglia output to superior colliuclus, one output neuron per co-ordinate (blue). Bottom: a spanning code created by basis functions, with one output neuron’s projection spanning one row or column (red). ***b***, Scaling of the number of controllable Cartesian co-ordinates by topographic or basis function mapping of basal ganglia output to superior colliculus. Topographic mapping is the best-case scenario of 1 neuron per co-ordinate. ***c***, Scaling of controllable co-ordinates with grid asymmetry. For a fixed number (50) of basal ganglia output neurons, the scaling of the number of controllable co-ordinates as the superior colliculus grid moves from a single row (ratio ∼ 0) to a symmetric grid (ratio = 1). ***d***, Simulations of basis function control of saccadic activity in superior colliculus. External input via the superficial layers specifies a saccade target in Cartesian co-ordinates (left), input to a 20 × 20 grid of collicular neurons middle. Twenty basal ganglia output neurons per side provide tonic inhibition of the superior colliculus: we plot here the inhibition received by each superior colliculus neuron (right). Unchanging inhibition prevents the build-up of saccadic activity at the target location (middle). ***e***, As for panel d, but now the basal ganglia neurons whose basis functions include the x-coordinates or the y-coordinates of the target location pause their firing, thus allowing at their intersection (right) the build up of saccadic activity (middle). ***f***, Two competing external inputs (left) could cause saccadic activity to increase at both locations in colliculus (middle), even if only a few basal ganglia output neurons paused their firing (right), because the second, upper target falls in the column covered by paused basal ganglia neurons whose basis functions are the y-coordinates. ***g***, As for panel f, but with other basal ganglia neurons increasing their firing and hence inhibition of collicular neurons (right, darkest rows), thus suppressing the build-up of saccadic activity (middle) at the second target location.

Less clear is how the basal ganglia output can provide that fine control over neural activity in the colliculus. The straightforward solution (Dominey and Arbib, 1992; Dominey et al., 1995; Jiang et al., 2003; Girard and Berthoz, 2005) is that the basal ganglia output also has a two-dimensional retinotopic map, and hence provides point-to-point control over collicular activity (Fig. 5*a*, top). But this scheme scales poorly because the number of possible co-ordinates scales linearly with the number of neurons (Fig. 5*b*, blue). And it seems at odds with the few neurons involved: of the basal ganglia output nuclei, only the substantia nigra pars reticulata (SNr) projects to the superior colliculus; that projection originates from at most two-thirds of the SNr (Deniau and Chevalier, 1992; McElvain et al., 2021); and even within that region the SNr neurons projecting to the superior colliculus are potentially in the minority, as antidromic stimulation of the colliculus activates far less than half of all sampled SNr neurons (Hikosaka and Wurtz, 1983c; Jiang et al., 2003).

The basis function idea provides a different solution, of the output neurons defining weights on basis functions tiling the two-dimensional plane. I give one example of how this solution might work; others are possible. In this *spanning code*, the projection of each basal ganglia output neuron (or group of) is a basis function that spans one row or one column of the two-dimensional co-ordinates for gaze direction. Then a particular co-ordinate is specified by the overlapping output of just two neurons (Fig. 5*a*, bottom).

The spanning code scales well, with the number of controllable co-ordinates rising quadratically with the number of neurons (Fig. 5*b*, red). As the spanning code uses just two output neurons to signal a particular location, its scaling is slower than a purely combinatorial code (Fig. 3*b*), and its scaling is slower when the grid is asymmetric (Fig. 5*c*). But it still scales better than standard theories of basal ganglia output to superior colliculus: for a given number of output neurons there are always more controllable co-ordinates for the spanning code than for the point-to-point wiring of a topographic map (Fig. 5*b,c*). So let’s check that the spanning code can indeed control collicular activity to provide appropriate motor commands for gaze direction.

Imagine a model where input specifying the target gaze direction (from e.g., the frontal eye fields or the superficial layer of the superior colliculus) arrives at a grid of intermediate layer collicular neurons (Fig. 5*d*; Methods). Activity at a location on that grid would represent a motor command to shift gaze to that target. At the same time, these neurons receive constant inhibitory input from a set of basal ganglia neurons, each of whose output spans rows or columns of the collicular grid as in Figure 5*a*. This constant inhibition suppresses all response to the target input (Fig. 5*d*), preventing a shift in gaze direction. Dropping the activity of basal ganglia output neurons whose projections intersect at the target location results in a hill of activity in the intermediate layer in neurons representing that location (Fig. 5*e*). Basis functions can thus allow suppression and selection of gaze direction changes.

This selection requires only a decrease in basal ganglia output, but I have been arguing that they encode bidirectional “dynamic weights”: what then might an increase in basal ganglia output encode here? One answer is to correct for unwanted loss of inhibition elsewhere on the two-dimensional map of gaze directions. Consider two competing target locations that lie on the same column (Fig. 5*f*, left): pausing intersecting basal ganglia outputs for one target could now result in a hill of activity at both target locations on the superior colliculus’ map (Fig. 5*f*, implying two simultaneous but different changes in gaze direction. However, increasing the output of basal ganglia neurons whose basis functions are the corresponding row of the unwanted target location will suppress the hill of activity at this location (Fig. 5*g*). Increasing basal ganglia output could ensure that at most one target location for gaze direction becomes active in the intermediate superior colliculus.

## Discussion

Basal ganglia output neurons are vastly outnumbered by their target neurons in the thalamus and brainstem. To this structural bottleneck is added the further dynamical bottleneck that this output is both constant and inhibitory. It is unclear how the basal ganglia’s output re-expands, both structurally and dynamically, to provide suitable control over its target regions.

I’ve offered as a solution the following idea: the activity of a basal ganglia output neuron defines the weight on a basis function defined by the pattern of its connections to its target regions. In this way, basal ganglia output can re-expand, for arbitrarily complex functions can in principle be constructed from the summation of a set of basis functions. The complex function approximated in this model is the level of inhibition across the target neurons of the basal ganglia output.

This model makes three predictions. First, output neurons all in their tonic state of constant firing do not necessarily imply uniform inhibition of their targets. Second, that both decreases and increases of output neuron firing are necessary to create the target function. Third, that individual output neurons will show low variation in their activity between identical events.

### Implications for our understanding of the basal ganglia

There are three main implications of these ideas and their predictions.

The first is that disinhibition is not the key to how basal ganglia output operates. Weights need to change in both directions to control basis functions, and create the desired target function by their summation. So under this account we’d expect basal ganglia output to both increase or decrease as necessary to set the desired target function of inhibition in its target. This provides a functional explanation for why basal ganglia output activity increases as well as decreases during movement or action (Gulley et al., 1999, 2002; Jin and Costa, 2010; Fan et al., 2012; Barter et al., 2015; Rossi et al., 2016; Schwab et al., 2020), which cannot be accounted for by classic disinhibition-based theories.

The second is that we can think of basal ganglia output as allowing rapid exploration of different activity patterns in a target region, which could be crucial in learning; for example of new actions such as skilled limb movements or songs. This is why I’ve called the basal ganglia output “dynamic weights” throughout, as simply by changing their activity the output neurons define a new function of inhibition in their target, which creates a different response in that target to the same input (Fig. 4).

The third is that basal ganglia outputs have no intrinsic “code”, but are control signals for a target region. The SNr has many apparent codes. Changes in SNr activity align to the onset of changes in gaze direction in monkeys that are stimulus or memory-driven (Hikosaka and Wurtz, 1983a,b; Handel and Glimcher, 1999, 2000; Basso and Wurtz, 2002; Sato and Hikosaka, 2002) and changes in movement in rodents (Lintz and Felsen, 2016). Jin and Costa (2010) report that SNr activity changes at the beginning and end of an action sequence, not at each action (though such discrete stop and start coding has not been found in striatum Sales-Carbonell et al., 2018). By contrast, Rossi et al. (2016) report SNr activity changes align to each individual lick a mouse makes on a spout. Fan et al. (2012) demonstrate that a pause in SNr activity can last for as long as a mouse holds down a lever, seemingly coding duration or a sustained action, rather than action onset or offset. And Barter et al. (2015) offer a startling demonstration of apparently continuous coding of a mouse’s head position in the (x,y) plane by the activity of individual SNr neurons, which typically coded either the x- or y-axis displacement of the head from its central position. Worse, as they are the bottleneck between striatum and the rest of the brain the basal ganglia output nuclei likely inherit other variables known to be encoded in striatum, and there are many of those, including time (Gouvêa et al., 2015; Mello et al., 2015), decision variables (Ding and Gold, 2013; Yartsev et al., 2018), possible actions (Klaus et al., 2019), their predicted value (Samejima et al., 2005; but see Elber-Dorozko and Loewenstein, 2018), and their kinematics (Rueda-Orozco and Robbe, 2015; Yttri and Dudman, 2016). How so few neurons could seemingly encode such a range of different variables is unclear. A starting point for a solution could be the dynamic weights theory offered here: basal ganglia output is not coding these variables, but are control signals for encoded variables in their target regions.

### Predictions for effects in target brain regions

The interpretation of basal ganglia output as encoding “dynamic weights” is a general principle for how that output can do useful work in its many target regions of the brain. Further specific predictions of this idea depend on the region targeted.

One prediction is that this re-expansion allows the basal ganglia output to shift cortical activity across an extensive repertoire of different states, via the shaping of thalamic output to cortex. The idea that basal ganglia output shifts the state or trajectory of cortical activity has been gaining traction: Athalye et al. (2020) looked at the problem of how motor cortical activity re-enters a desired state after a reward, and proposed a conceptual model for how plasticity at cortico-striatal synapses may alter basal ganglia output to shift cortical activity; Logiaco et al. (2021) looked at the problem of how motor cortex can drive sequences of movements, developing and analysing a model of how shifting basal ganglia output can switch motor cortical activity states and thus create movement sequences (though based only on disinhibition setting thalamic units on or off). Which regions of cortex this prediction extends too is unclear; speculatively, it would be in the layers (II/III and V) of cortical regions that receive direct input from the motor and intralaminar thalamic nuclei that the basal ganglia output directly influences (Nishimura et al., 1997; Kha et al., 2001; Middleton and Strick, 2002; Bodor et al., 2008; Kuramoto et al., 2011). This prediction is consistent with data from primates (Sauerbrei et al., 2020), rodents (Inagaki et al., 2022) and songbirds (Moll et al., 2023) showing that motor thalamic input to motor cortex is necessary for initiating, or changing to, a discrete element of movement.

It follows that manipulating the state of basal ganglia output projections to thalamus should change the state of cortical activity, and hence alter behavior. Recent studies that optogenetically manipulated SNr axon terminals in motor thalamus reported that inhibiting these SNr terminals during a licking task biased licking to the contralateral side, whereas activating these terminals stopped licking all together (Morrissette et al., 2019) or prevented impulsive licking (Catanese and Jaeger, 2021). These data are consistent with shifting the state of cortical activity; but they are also consistent with classic disinhibition ideas that pauses in basal ganglia output are a go signal and increases in the output are a stop signal. Such optogenetic stimulation that broadly targets all terminals with a stereotyped pattern of stimulation is repeatedly setting all the dynamic weights to approximately the same values. Distinguishing classic disinhibition and dynamic weight accounts of basal ganglia output would instead need selective stimulation of non-overlapping sets of SNr terminals: the dynamic weights idea predicts this would result in different states of cortical activity, and hence potentially different behavioral responses.

If the basis functions defined by the connections of basal ganglia output neurons have some topographic organization, as in the example of superior colliculus, then different predictions arise for manipulating their activity. Selective optogenetic activation of a few SNr neurons that project to the superior colliculus would be predicted to maximize the weight on their basis functions, strongly inhibiting a region of the intermediate superior colliculus, and so create a region of visual space that it is difficult to shift gaze or orient towards. The shape of that region would depend on the shape of the basis functions. The specific spanning code advanced here (Fig. 5) would predict that the region of visual space would cover entire x- or y- co-ordinates. This prediction is consistent with reports that the activity of individual SNr neurons encodes the entire x-axis or y-axis displacement of the head in mice (Barter et al., 2015). That said, as noted above the spanning code is not an optimal form of basis function tiling, and other tilings of the collicular representation of gaze direction, including random projections, would be worth further exploring.

### What the dynamic weights idea does not yet address

There are a few key issues that further development of this “dynamic weights” idea could usefully address. The first is its precise role in behavior. Most current basal ganglia theories focus on how its output controls action, but fall largely into two opposing camps. One is that basal ganglia output controls action selection, via pauses in its tonic inhibition (Redgrave et al., 1999; Bogacz and Gurney, 2007; Liénard and Girard, 2014; Klaus et al., 2019); the other is that basal ganglia output controls action specification, by its reduced activity modulating some aspect of the kinematics or gain of movement (Turner and Desmurget, 2010; Park et al., 2020; Thura et al., 2022). The dynamic weights idea is currently agnostic to these theories: indeed, it suggests that coding is in the target region, not in basal ganglia output per se. Changes in cortical state by basal ganglia output could be the selection of action or change in the kinematics of an action. Similarly, the example given of superior colliculus control follows previous models of SNr output to colliculus (Girard and Berthoz, 2005) in interpreting the hill of activity as selecting an action, the new direction of gaze; but one could interpret this as specifying action, setting the velocity of the change in gaze direction by controlling the size of the hill of activity.

Both canonical action selection (e.g., Gurney et al., 2001; Frank, 2005; Humphries et al., 2006; Liénard and Girard, 2014; Lindahl and Hellgren Kotaleski, 2016) and specification (e.g., Yttri and Dudman, 2016; Park et al., 2020) theories also assume basal ganglia output are organized as a collection of parallel populations or “channels,” each one representing an option to be selected or specified. While this assumption is the basis for powerful explanations of how the basal ganglia circuit can implement selection or specification, it also strongly restricts the coding capacity of the basal ganglia output. The theory here is as yet agnostic to the size of the basal ganglia output population **a** over which a target function **f** could be specified. Given the broadly overlapping targets of neighboring SNr neurons (Deniau and Chevalier, 1992) there could be just one population whose goal is to create a specific trajectory of activity in its targets (Wärnberg and Kumar, 2023).

A second issue is of how control of the dynamic weights can be learnt. The theory outlined here assumes the connections made by the basal ganglia output nuclei have fixed strengths (Fig. 2), defining the basis functions, and it is the activity of the output neurons that set the weights of those functions. Unlike traditional views of basis functions with weights that are learnt, these dynamic weights can be adjusted on the fly by input from upstream nuclei of the basal ganglia. Learning when and how to adjust them becomes a question of how feedback from the environment can change the input to the output nuclei. An obvious answer is that environmental feedback is the prediction error signal conveyed by phasic dopamine release in the striatum (Schultz et al., 1997; Bayer and Glimcher, 2005; Rutledge et al., 2010; Hart et al., 2014; Howe and Dombeck, 2016); and that this modulates plasticity at cortico-striatal synapses (Reynolds et al., 2001; Shen et al., 2008; Gurney et al., 2015) to change the input to the basal ganglia’s output nuclei. Extending the theory here to include explicit striatal input and cortico-striatal plasticity would be an interesting line of work.

That striatally targeted dopamine acts like a reward prediction error and modulates cortico-striatal plasticity are key reasons why the basal ganglia are often viewed as a central part of the brain’s distributed system for reinforcement learning (Ito and Doya, 2011). In this view, the value of a state or action is represented in the cortex, cortico-striatal connection weights, or striatal activity (Houk et al., 1995; Joel et al., 2002; Khamassi et al., 2005; Samejima et al., 2005; Bogacz and Larsen, 2011; Collins and Frank, 2014; Blackwell and Doya, 2023) [see also Elber-Dorozko and Loewenstein, 2018 for dissenting evidence], and the downstream basal ganglia circuit enact policy by selecting (Frank, 2005; Humphries et al., 2012; Collins and Frank, 2014) or specifying (Yttri and Dudman, 2016) an action based on that value. As reinforcement learning algorithms place no constraints on how policy is implemented, this view is silent on *how* the basal ganglia circuit selects or specifies action, and silent on the existence of the bottleneck and of any solutions to it. Some forms of reinforcement learning use basis functions to approximate a continuous space of states or actions (Sutton and Barto, 2018). This use of basis functions is distinct from the ideas offered in this paper: here, the basis functions are a property of anatomy, not abstract quantities, and are not explicitly representing anything. Indeed, all theories mapping elements of reinforcement learning algorithms to the basal ganglia could turn out to be false, but if they were false it would not affect the ideas in this paper. The converse may not hold: an open question is whether the re-expansion of basal ganglia output places any limits on mappings of reinforcement learning algorithms to the basal ganglia circuit.

Finally, while the dynamic weights idea provides a solution to the problem of the computational bottleneck, it does not explain why this bottleneck exists. Some ideas seem worth pursuing here. Producing spikes makes up much of a neuron’s energy demand (Attwell and Laughlin, 2001; Laughlin, 2001; Sengupta et al., 2010), and so if the high activity rates of the output nuclei are necessary to their function, this could place energetic constraints on their size. Such bottlenecks can also occur as networks evolve when the mapping between their input and output has fewer dimensions than either of the input or the output itself (Friedlander et al., 2015). But, however it arose, the computational bottleneck of basal ganglia output provides a strong challenge to any theories for functions of the basal ganglia.

## Materials and Methods

### Code availability

All MATLAB code is available under a MIT License from https://github.com/mdhumphries/Basal_Ganglia_Bottleneck_simulations. The code is also available in Extended Data 1.

10.1523/ENEURO.0431-23.2024.d1 Extended Code Download Extended Code, ZIP file.

### Estimating the expansion of basal ganglia output

Targets of the mouse SNr were accessed from the Allen Mouse Brain Atlas (Oh et al., 2014) at https://connectivity.brain-map.org/. The Atlas contains seven experiments that had the injection of a fluorescent anterograde tracer targeted to the SNr of the right hemisphere. One experiment (ID:3035444) filled a volume (0.04 mm^3^) an order of magnitude smaller than the others, occupying less than 3% of the SNr, and so was not used here. Table 1 gives data on the six retained experiments.

**Table 1.** Allen mouse Brain Atlas experiments

ID	Volume injected (mm^3^)	Proportion in SNr	Proportion of SNr injected
478096249	0.49	0.41	0.30
158914182	0.54	0.29	0.23
478097069	0.43	0.32	0.21
100141993	0.23	0.95	0.32
299895444	0.36	0.94	0.51
175263063	0.17	0.96	0.24

The proportion of each injection that fell in the SNr was manually scraped from each experiment’s webpage at https://connectivity.brain-map.org/. Proportion of SNr injected used the SNr volume of 0.69 mm^3^ from the Blue Brain Cell Atlas.

Each experiment contained a complete list of all regions in the Allen Common Coordinate Framework V3.0 that had tracer found in them, in both hemispheres, and the volume and density of fluorescent pixels in each region. The Allen Common Coordinate Framework follows a hierarchy from the whole brain, through the gray matter, down to fine-grained subdivisions of nuclei: identified target regions thus overlapped with, or were subdivisions of, others. To find a single, non-overlapping set of targets, I used the list of 295 non-overlapping gray matter regions from Oh et al. (2014), their Supplementary Table 1.

A complete set of neuron numbers for each of those regions in the mouse brain was obtained from Blue Brain Cell Atlas dataset published in Rodarie et al. (2022), their S2 Excel file.

With these data to hand, for each experiment I did the following:

1. Found all targets within the list of 295 unique regions, in both hemispheres
2. Rejected any target region that had a fluorescent pixel density less than some threshold *θ*
3. Looked up the neuron count *n*_*t*_ in each target region *t* from the Blue Brain Cell Atlas
4. Computed the total potential SNr connections: summed neurons across all *N* retained target regions $\sum^{N}n_{t}$
5. Computed an estimate of SNr connections by estimating axonal arborization in each target: weighted the neuron count in each region by its density *d*_*t*_ ∈ [0, 1) of fluorescent tracer $\sum^{N}n_{t}\times d_{t}$.

**Figure 6.**
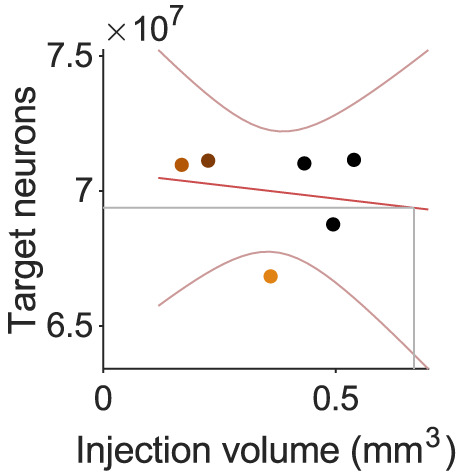
The need for a threshold for noise in tracing experiments. Here, I plot the total number of target neurons when including every target region of the SNr with detectable fluorescent pixels in the Allen Mouse Brain Connectivity Atlas experiments. As all or almost all regions had detectable pixels, the total number of neurons is at or close to the total number of neurons in the mouse brain. Also note how the number of neurons does not scale with the injected volume.

Thresholding by the density of fluorescence within each target region (step 2) eliminated image noise and other artefacts: without a threshold, all 295 regions contained fluorescent pixels implausibly suggesting the SNr projected to the entire brain (Fig. 6).

Linear models were fit to the scaling of the total number of target neurons (step 4) and of the estimate of SNr connections (step 5) with the volume of injection in each experiment (fitlm, MATLAB2023a). Extrapolating these models to the volume of the SNr (predict, MATLAB2023a) gave upper and lower bounds on the total number of SNr target neurons. From these were computed upper and lower bounds on the ratio of target to SNr neurons. Computing the upper and lower ratio bounds for a range of density threshold *θ* between 0 and 0.05 in steps of 0.005 revealed they reached an asymptote with increasing *θ* (Fig. 1*c,d*) implying a stable estimate. The asymptote itself was detected as three consecutive bound estimates that changed by less than 1, and was reported as the final of these estimates.

As Table 1 shows, three of the experiments had injections that considerably exceeded the bounds of the SNr. Robustness to this limitation was tested in two ways. In the first, the linear model was fit weighting the data points by the proportion of SNr filled in each experiment: this gave the same results for the asymptotic ratios as the unweighted linear model.

In the second, I separately considered each of the three experiments with more than 90% of their injection within the SNr. For each experiment, I first computed the estimated total number of target neurons and the estimate of SNr connections (steps 4 and 5 above). I then computed the estimated number of SNr neurons $\hat{S}$ within the injection volume, given by

$$\hat{S}=\#\;\mathrm{neurons}\;\mathrm{in}\;\mathrm{SNr}\times \frac{\mathrm{Injection}\;\mathrm{volume}}{\mathrm{Volume}\;\mathrm{of}\;\mathrm{SNr}},$$

and finally computed the ratio of target neurons to $\hat{S}$. The resulting upper and lower bound ratios of target to SNr neuron numbers also reached an asymptote for each of three experiments (Fig. 1*e,f*). The asymptote was computed as above separately for each experiment; the mean of these is reported.

### Using basis functions for expansion

My proposal here stems from the use of basis functions to approximate a target function, so I give a brief account of that here to make the analogy clear.

The target function is *f*(*x*), which can be of any dimension (for example, I use one-dimensional functions in Figs. 2 and 4, and two-dimensional in Fig. 5). In the most general form of approximating *f*(*x*), we choose some basis function *g*(*x*, Ω), which is defined on some interval *x* and takes parameters Ω, and we obtain the approximation $f^{\prime }(x)$ of *f*(*x*) by

$$f^{\prime }(x)=\sum_{i=1}^{B}w_{i}g(x,\Omega_{i}),$$

the sum of *B* such basis functions each parameterized by Ω_*i*_.

Approximating a function typically uses the radial basis functions *g*(*x* − *c*, *σ*), which are symmetric about their center *c* and have a single parameter *σ* that defines their width. Gaussians are one type of this class of functions, as shown in Figure 2*a*. The approximation of *f*(*x*) is then

$$f^{\prime }(x)=\sum_{i=1}^{B}w_{i}g(x-c_{i},\sigma ),$$

the sum over a set of *B* such basis functions, placed at centers *c*_1_, *c*_2_, …, *c*_*B*_, and each with its own weight *w*_*i*_. Approximating *f*(*x*) then depends on choosing *g*(*x*), setting an appropriate number *B* of these, and finding appropriate weights to minimize some error function $e(f(x)-f^{\prime }(x))$. When using basis functions to fit curves (in one dimension) or surfaces (in two or more dimensions) to sampled data, typically the number and location of sampled data-points respectively determines *B* and the set of *c*_*i*_.

Expansion occurs because we want the target function *f*(*x*) to be defined over *n* points in one dimension (or *n* × *m* points in two dimensions, and so on), and typically *B* ≪ *n*. This is especially true when using basis functions to approximate functions across a full range of *n* points using just *B* sampled data points.

### The basal ganglia output as a set of dynamic weights

The theory outlined here is that *g*(*x*) is realized by the strength distribution of an output neuron and the weights *w*_*i*_ are defined by the activity of that neuron (or equivalently, the strength distribution of a group of output neurons taken collectively, and their collective activity). Then $f^{\prime }$ is the inhibition function defining the input to every neuron in the target nucleus.

To make this more concrete, consider that there are *b* basal ganglia output neurons that project to target region(s) containing *n* neurons, with *b* ≪ *n*. Then expressing the basis function idea in matrix form, we get:

Da=f,

where **a** is the *b*-length vector of basal ganglia output activity, **f** is the *n* length vector of inhibition arriving at the target neurons–the target function in Equation 4–and **D** is the *n* × *b* matrix that defines the basis functions. For example, for a one-dimensional set of overlapping basis functions that define a ring (cf Fig. 4*a*), each column of **D** is a basis function, shifted circularly across columns.

If the columns of **D** are each a circularly shifted version of the same basis function **v**, and no column can be identical, then the construction of **D** is:

$$D=\sum_{i}^{b}a_{i}⊗v_{i},$$

where **v**_*i*_ is the *i*th shifted version of **v** and **a**_*i*_ is a vector with a 1 at the *i*th entry and 0s at all others. Consequently, Equation 6 defines a full rank matrix, i.e., its columns are linearly independent.

### Recurrent neural network modelling of cortex

The recurrent neural network model of cortex contained *N* = 200 units, 80% excitatory and 20% inhibitory, whose dynamics were given by:

$$\tau \frac{dx}{dt}=-x+Wr(x)+I(t),$$

where **x** is the *N*-length vector of unit potentials, *τ* = 20 ms is the network’s time constant, **W** is the *N* × *N* connectivity matrix, *r*() is a function that defines each unit’s firing rate, and **I**(*t*) is an *N*-length vector of inputs to each unit at time *t*. I used here the stabilized supra-linear network (Ahmadian et al., 2013; Rubin et al., 2015; Hennequin et al., 2018) in which the firing rate of the *i*th unit was given by the rectified power-law,

$$r(x_{i})=k\lfloor x_{i}\rfloor^{\eta },$$

with *k* = 0.1 and *η* = 2. Numerical simulations used forward Euler with a time-step of 0.1 ms.

Random connectivity matrices **W** of size *N* × *N* were created by connecting units with probability *p* = 0.1, then randomly assigning columns to be excitatory or inhibitory units. Weights of connections in a column were then defined as $w_{0}\;/\;\sqrt{N}$ for excitatory connections and $\left(-\gamma w_{0})\;/\;\sqrt{(}N\right)$ for inhibitory connections, where $w_{0}=R\;/\;\sqrt{\;p(1-p)(1+\gamma^{2})/2}$, the E:I weight ratio *γ* = 3, and the spectral radius *R* = 10 (Rajan and Abbott, 2006; Hennequin et al., 2014). I tested versions with and without further stability optimization of the resulting random matrix **W**; further optimization used the algorithm of Hennequin et al. (2014) that minimizes the maximum real eigenvalue of **W**, resulting in networks whose dynamics recapitulate those of motor cortex (Hennequin et al., 2014; Rodriguez et al., 2024). Similar results were obtained in both random and optimized **W**; results from a random network are plotted in Figure 4. Similar results were also obtained in networks with a 50:50 ratio of excitatory and inhibitory units (not shown).

The vector **I**(*t*) comprised inputs from unmodeled regions of cortex and from thalamus. Background input of **I**(*t*) = 0.1 was applied for *t* < 1,000 and *t* > 2,000. For time 1,000 ≤ *t* ≤ 2,000 the inputs stepped up or down from background.

The stepped thalamic input to cortex was assigned to *n* = 0.1*N* randomly chosen entries of **I**(*t*). It was modeled as the vector **T** whose *n* entries were given by **T** = *T*_*b*_ − **Da**, where *T*_*b*_ = 1 was baseline thalamic activity, **a** was the *b* = 0.25*t* length vector of basal ganglia output activity randomly chosen in [0, 0.4], and **D** was the *n* × *b* matrix of connection weights from basal ganglia output to thalamus. Each column of **D** was thus the basis function defined by each basal ganglia output neuron’s projections to thalamus: here each basis function was symmetric with 1 at the center and steps down of 0.2 on either side; the basis functions were circularly shifted to uniformly tile the matrix (Fig. 4*a*).

The stepped cortical input was modeled by the remaining 0.9*N* entries in **I**(*t*), which were randomly assigned an initial value in [0, 1]. The stepped thalamic and cortical input were then held constant for 1,000 ≤ *t* ≤ 2,000.

When simulating the effects of changing basal ganglia output, only the entries of **a** were resampled in each simulation, with all other stepped inputs **I**(*t*) sampled once and then held constant across simulations.

### Superior colliculus grid model

The build-up layer of the superior colliculus was modeled as a *N* × *N* grid of units, with *N* = 20 here. The dynamics of units on the grid were given by

$$\tau \frac{ds}{dt}=-s+u(t)+Da,$$

where **s** was the *N* × *N* length vector of collicular unit activity and *τ* = 10 ms was the unit’s time constant. Inputs specifying the saccadic target location were represented in the *N* × *N*-length vector **u**. Input from *b* = 2*N* basal ganglia output neurons was defined by the *b*-length vector of their output **a** and the (*N* × *N*) × *b* matrix **D** that defined the basis functions. The entries of **D** were −1 or 0, set so that each output neuron connected to either an entire row or column of the collicular grid (Fig. 5*a*, bottom).

Colliculus output activity plotted in Figure 5 is given by the rectified linear function r=max(0,s).

## Appendix: Limits on the number of thalamic neurons reachable by basal ganglia output

The model of basal ganglia output to thalamus adopted in the main text (Fig. 4*a*) gives some interesting insights into potential constraints on the divergence of basal ganglia projections to thalamus.

In the model, each of the *b* basal ganglia output neurons has a symmetric distribution of output strengths (Fig. 4*a*, inset), which defines its basis function. Given the number of possible discrete strength values *N*_*b*_, the width of this distribution is *N*_*T*_ = 2*N*_*b*_ − 1, which defines the number of thalamic neurons reachable by one basal ganglia output neuron.

Each basal ganglia output strength distribution is centred on a specific thalamic neuron *t*_*i*_. Let’s assume the distributions tile the thalamus in a one-dimensional ring to avoid edge effects, and the centres of the distributions are spaced the same number *d* of thalamic neurons apart. The number of possible thalamic neurons in this ring model is then *T* = *d* · *b*.

If we want every thalamic neuron to receive basal ganglia input, then the maximum distance *d* between the centres of the distributions must also be the width of the distributions: *d*_max_ = 2*N*_*b*_ − 1. Consequently, the maximum number of thalamic neurons must be *T*_max_ = (2*N*_*b*_ − 1)*b*.

But if the basal ganglia output distributions are spaced at distance *d*_max_ then each thalamic neuron receives only one basal ganglia input, and so the basis functions are not overlapping. The distance between basal ganglia output distributions must therefore be *d* < *d*_max_. How much smaller would depend on the minimum desired number of overlapping basal ganglia inputs to each thalamic neuron.

Table 2 gives the minimum number of overlapping basal ganglia output neurons in this ring model, for a range of distances *d* between, and resolutions *N*_*b*_ of, the basal ganglia output strength distributions. For example, with the centres of the distributions spaced *d* = 3 thalamic neurons apart, and the distributions having *N*_*S*_ = 6 possible strength values, each thalamic neuron receives input from at least three basal ganglia output neurons.

**Table 2.** **Minimum number of basal ganglia inputs to one thalamic neuron**. Each entry is the minimum number of inputs each thalamic neuron receives at the specified strength resolution (*N*_*b*_) and distance *d* between strength distributions. These were numerically enumerated, using *b* = 2 · max*N_b_*

	*N*_*b*_													
*d*	3	4	5	6	7	8	9	10	11	12	13	14	15	16
1	5	7	9	11	13	15	17	19	21	23	25	27	29	31
2	2	3	4	5	6	7	8	9	10	11	12	13	14	15
3	1	2	3	3	4	5	5	6	7	7	8	9	9	10
4	1	1	2	2	3	3	4	4	5	5	6	6	7	7
5	1	1	1	2	2	3	3	3	4	4	5	5	5	6
6	0	1	1	1	2	2	2	3	3	3	4	4	4	5
7	0	1	1	1	1	2	2	2	3	3	3	3	4	4
8	0	0	1	1	1	1	2	2	2	2	3	3	3	3
9	0	0	1	1	1	1	1	2	2	2	2	3	3	3
10	0	0	0	1	1	1	1	1	2	2	2	2	2	3

We see that the minimum number of inputs falls rapidly with increasing distance between distributions. If the distance is greater than 4, then increasing the minimum number of inputs above 2 or 3 requires a large increase in the possible number of strength values. What then should *d* and *N*_*b*_ be?

A conservative lower bound on the estimated ratio of thalamic to basal ganglia output neurons is on the order of 5:1 (Figure 1). As the number of thalamic neurons in our ring model here is *T* = *d* · *b*, so this ratio implies *d* = 5. And with that distance between strength distributions, that implies a strength resolution of at least *N*_*b*_ ≥ 11 for each thalamic neuron to receive input from at least four basal ganglia output neurons.

## Synthesis

Reviewing Editor: Arvind Kumar, KTH Royal Institute of Technology Decisions are customarily a result of the Reviewing Editor and the peer reviewers coming together and discussing their recommendations until a consensus is reached. When revisions are invited, a fact-based synthesis statement explaining their decision and outlining what is needed to prepare a revision will be listed below. The following reviewer(s) agreed to reveal their identity: NONE.

Synthesis

First of all, apologies for the delay in getting the reviewers' feedback and then reaching a decision. One of the reviewers has extenuating circumstances which delayed the process.

Both the reviewers think that the idea to think of the basal ganglia function to control the weights of basis function makes sense. But this perspective is not really new because RL pretty much does that. And the basis function formulation of RL is already there in the text books (Sutton and Barto 2nd edition) and in the very first paper that linked dopamine to RL (Schulz et al. 1998). It may be true that no one has explicitly stated this in the context of basal ganglia, therefore, this paper may be interesting.

But it is important to make the model more specific. For instance, reviewers would like you to go beyond the 'proof of concept' style. Author's database analysis of basal ganglia anatomy is also rather superficial.

With regard to the prediction reviewers think that the prediction prediction about the trial-by-trial variability is interesting but that may not follow from the basis function idea. And the other two predictions are extensions of the definitions. SO this aspect of the model needs a careful revision.

So overall we think that the manuscript needs a thorough revision. In the following I append the comments from the two reviewers which can further help to revise the manuscript.

Reviewer #1

This manuscript theoretically proposes a view that the basal ganglia output operates as dynamic weights of basis functions, thereby enabling a variety of roles in action/motor control etc despite its limited capacity (number of neurons) compared to the input structure, i.e., striatum (the "bottleneck" structure). The authors present this theory using an elementary linear system model, and then extend it to via-thalamic control of cortical states (how the relatively small number of thalamic outputs can control rich cortical dynamics) and via-superior colliculus control of eye movements (how the small number of SNr neurons can control flexible eye movements).

I think the authors' view that the basal ganglia operate as controlling the weights of basis functions is quite reasonable, and would not have strong objections to it. However, at the same time I am afraid that it may not be really new. A dominant theory of basal ganglia function is the reinforcement learning (RL), which is not mentioned in this manuscript (I think it is problematic), and in this RL theory it has long been a standard notion to represent states or actions by a set of features and approximate their values by updating the weights over these features (basis functions). Indeed, it was already well formulated in the first edition of popular RL textbook by Sutton and Barto back in 1998. And importantly, there have been quite a few studies that explicitly or implicitly assumed and/or suggested that such value function approximation using basis functions is implemented in the cortico-basal ganglia-dopamine system. Some recent examples can be seen in the second edition of the Sutton and Barto RL textbook (2018).

The authors might argue that the RL theory only describes a reward/value-learning function of basal ganglia while this manuscript more generally deals with basal ganglia functions. However, in fact the RL theory can in fact describe a wide range of learning and action selection / decision making behavior. For example, basal ganglia control of eye movement has been much studied in the RL context.

An important limitation of the authors' model is that how the dynamic weights of basis functions can be appropriately learnt/adjusted. Without such learning, even though the system can have a large representational power, it cannot achieve meaningful computations. Regarding this point, again the RL theory has provided an elegant solution, where the weights of basis functions can be updated using the temporal-difference prediction errors, and it appears to match a wealth of biological observations that dopamine signals appear to encode such prediction errors.

Another important issue is that whereas the authors considered the bottleneck from the striatum to the basal ganglia output, another (and possibly even more prominent) convergence occurs at the cortico-striatal projections: inputs from a wide cortical regions (and also other regions such as amygdala) to the relatively compact striatum would need to be considered as a part of the bottleneck structure. Indeed, many RL theories assume this convergence and consider that the cortex represents states or actions by features while the striatum represents the weights of their values (while RL theories may typically not consider the within-basal-ganglia convergence from the striatum to the output nuclei - this point could be a good feature of the authors' model if it is more emphasized and developed in comparison with the RL models).

If the authors want to claim that their model is conceptually different, and/or superior to, the RL theory-based models of basal ganglia, I think it is necessary to cite these RL models and explain how the authors' model exceeds, or can stand in parallel with, these previous models. The authors' style of introducing the view of basal ganglia as dynamic weights of basis functions may be good for tutorial-type documents, or potentially perspective-like articles, but if the authors aim at original research article, I think the abovementioned issues need to be resolved.

<additional points>

Line 32-33 "a lengthy list of proposed functions"

Even if the author does not intend to make this list of proposed functions comprehensive, value / reward learning should be one of the most suggested roles of basal ganglia, and so naming it with some citations is very recommended.

Line 65

Is it really the case that mice have more output neurons than rats have? Or maybe it depends on how to count the number of cells. Anyway please do a double check.

Figure 1

Do the areas of the circuits in this figure represent the numbers of neurons? Good to explain in the caption.

Line 93-102

I understand that the binary view may be too pessimistic about the information capacity of basal ganglia, but I could not understand why the author thinks dis-inhibition generally limits information coding. Dis-inhibition does potentially cause energy consumption issue (constant firing of output neurons should consume much energy), but other than that, is it basically just a reversal of sign? And indeed, it seems this point is not considered as a major limitation in many value learning theories of basal ganglia.

Line 217 and descriptions around there

Arguments about linear system appears not really relevant, because neural systems are very unlikely to be linear (even though I agree that starting from linear system would be good for understanding in many cases, and I actually like this kind of introduction in textbook or tutorials).

Line 494

It is OK to assume that all the neurons, rather than only a part of them, were labeled by tracer?

Line 495

Is there anything that can justify the "staining density threshold of 5% in the target region"? What occurs if this threshold changes, e.g., to 1%?

Reviewer #2

The manuscript "The computational bottleneck of basal ganglia output (and what to do about it)" begins with quantifying (using the Allen Brain Atlas and the Cell Atlas) that the output nuclei of the basal ganglia (SNr and GPi/EP) target a larger set of downstream neurons than are present in these nuclei themselves. This, the author argues, creates a computational bottleneck in these nuclei. Their proposed solution is to reconsider the representation in these nuclei and think of them as weights for a set of basis functions activating downstream nuclei, in contrast to either a homogeneous go/no-go signal or a discrete action selection. The author then explains the concept of basis functions in some detail and provides some examples of how it may be used to understand the basal ganglia.

Overall, I found the paper well-written, and I believe the detailed build-up of the model will help readers without formal mathematical training to follow the argument. Furthermore, I agree with the case of BG representing basis functions, and I think the author both explains this idea well and cites much of the key literature in support of it.

On a first reading of the manuscript, my main concern was how unexpected and novel this argument really is. That is, my first guess would be that if we assume the gradual increases and decreases in firing rates of individual GPi/EP/SNr neurons are computationally relevant, they can likely be interpreted as weights of basis functions. The only real alternative I can think of is completely isolated channels (so that the basis functions are one-hot unit vectors), but that seems unlikely from an anatomical point-of-view (as also pointed out in the manuscript). Hence, my reading of much of the "action specification" literature (e.g. Park et al. 2020 cited in the manuscript, and Fig 1d of Dhawale et al. 2021, Nat Neuro, https://doi.org/10.1038/s41593-021-00889-3) have been that they are arguing for some kind of basis-function model. But on a second reading of the manuscript, I am now more convinced that the precise argument has not been (this well) spelled out before. Indeed, the closest treatment I can recall reading is Gershman &Uchida (2019; http://dx.doi.org/10.1038/s41583-019-0220-7), but in contrast to the present manuscript, Gershman &Uchida argue that the striatum uses basis functions to represent a probability distribution of the current state, which is quite different from using basis functions to represent the output(s) of the basal ganglia. Therefore, I think the manuscript would be a valuable addition to the literature.

That said, I have a few suggestions and questions:

* For Figure 4, I think it would also be nice to somehow illustrate that the same control of cortical dynamics could not be achieved without using basis functions in the output of BG. That is, is it not possible to control cortex to a similar degree with completely non-overlapping/"one-hot" connections from BG to thalamus, or with homogeneous all-to-all connections?

* The model of how basal ganglia control cortical dynamics (Figure 4) could be related to a similar model proposed by Athalye et al. (2020, https://doi.org/10.1016/j.conb.2019.11.023). There are also some experimental results from the same authors that may provide some tentative experimental support of the model in the manuscript.

* The discussion about completeness/degeneracy of the output matrix D (lines 199-241) could be complemented with a more dynamical perspective of essentially the same problem, namely by considering the controllability (in the control-theoretic sense) of the downstream areas rather than the number of unique patterns that could evoke a particular output (see Kao &Hennequin, 2019, https://doi.org/10.1016/j.conb.2019.09.001). A control-theory perspective, rather than just a linear algebra one, might also help connect Figure 3 (which is very much static coding) with Figure 4 (which is about dynamics).

* Why does the RNN that model cortex have 50% inhibitory neurons?

* I am not fully convinced about the argument in Figure 5. The basis functions can only be more efficient than one-to-one connections if there is some assumption of smoothness on the code that is matched with the shape/metric of the basis functions. As a very contrived example, say that the subject is watching a chessboard (8x8 black-and-white squares) and is by training expecting a cue to appear in any of the white squares, but not any of the black ones. Wouldn't we then expect the BG to send a chessboard pattern to the SC under the model proposed in the Figure 5? With the column/row coding proposed there, I think one would need as many basis functions as there are squares, and thus in this particular example they are no more effective than a one-to-one mapping. Of course, in most realistic scenarios, basis coding will be more efficient, but this is due to better compliance with (smoothness) assumptions, and not pure mathematics. In the figure and text, one might get the impression that basis functions are mathematically guaranteed to always be better, which they are not.

* On lines 387-408, the author argues that basis functions can reconcile disparate findings in the experimental literature, but I cannot follow how all the examples that are listed could be reconciled simply by considering BG output as basis functions. For example, how do basis functions help explain why some investigators find chunking/bracketing and some find more-or-less uniform tiling? What would bracketing mean in terms of basis functions? And why exactly would SNr/GPi/EP basis functions be better for all the various striatal codes listed? Are there basis functions also in the striatum?

* For outlook in discussion: is a basis function representation unique for the SNr/GPi/EP which have a bottleneck, or is it an ubiquitous coding scheme of the basal ganglia/rest of the brain?

## References

[B1] Ahmadian Y, Rubin DB, Miller KD (2013) Analysis of the stabilized supralinear network. Neural Comput 25:1994–2037. 10.1162/NECO_a_00472 23663149 PMC4026108

[B2] Anderson RW, Keller EL, Gandhi NJ, Das S (1998) Two-dimensional saccade-related population activity in superior colliculus in monkey. J Neurophysiol 80:798–817. 10.1152/jn.1998.80.2.798 9705470

[B3] Athalye VR, Carmena JM, Costa RM (2020) Neural reinforcement: re-entering and refining neural dynamics leading to desirable outcomes. Curr Opin Neurobiol 60:145–154. 10.1016/j.conb.2019.11.023 31877493

[B4] Attwell D, Laughlin SB (2001) An energy budget for signaling in the grey matter of the brain. J Cereb Blood Flow Metab 21:1133–1145. 10.1097/00004647-200110000-00001 11598490

[B5] Barter JW, Li S, Sukharnikova T, Rossi MA, Bartholomew RA, Yin HH (2015) Basal ganglia outputs map instantaneous position coordinates during behavior. J Neurosci 35:2703–2716. 10.1523/JNEUROSCI.3245-14.2015 25673860 PMC4323537

[B6] Basso MA, Sommer MA (2011) Exploring the role of the substantia nigra pars reticulata in eye movements. Neuroscience 198:205–212. 10.1016/j.neuroscience.2011.08.026 21884760 PMC3314308

[B7] Basso MA, Wurtz RH (2002) Neuronal activity in substantia nigra pars reticulata during target selection. J Neurosci 22:1883–1894. 10.1523/JNEUROSCI.22-05-01883.2002 11880518 PMC6758865

[B8] Bayer HM, Glimcher PW (2005) Midbrain dopamine neurons encode a quantitative reward prediction error signal. Neuron 47:129–141. 10.1016/j.neuron.2005.05.020 15996553 PMC1564381

[B9] Blackwell KT, Doya K (2023) Enhancing reinforcement learning models by including direct and indirect pathways improves performance on striatal dependent tasks. PLoS Comput Biol 19:e1011385. 10.1371/journal.pcbi.1011385 37594982 PMC10479916

[B10] Bodor AL, Giber K, Rovó Z, Ulbert I, Acsády L (2008) Structural correlates of efficient GABAergic transmission in the basal ganglia-thalamus pathway. J Neurosci 28:3090–3102. 10.1523/JNEUROSCI.5266-07.2008 18354012 PMC2670451

[B11] Bogacz R, Gurney K (2007) The basal ganglia and cortex implement optimal decision making between alternative actions. Neural Comput 19:442–477. 10.1162/neco.2007.19.2.442 17206871

[B12] Bogacz R, Larsen T (2011) Integration of reinforcement learning and optimal decision-making theories of the basal ganglia. Neural Comput 23:817–851. 10.1162/NECO_a_00103 21222528

[B13] Bryden DW, Johnson EE, Diao X, Roesch MR (2011) Impact of expected value on neural activity in rat substantia nigra pars reticulata. Eur J Neurosci 33:2308–2317. 10.1111/j.1460-9568.2011.07705.x 21645133 PMC3334837

[B14] Buhusi CV, Meck WH (2005) What makes us tick? Functional and neural mechanisms of interval timing. Nat Rev Neurosci 6:755–765. 10.1038/nrn1764 16163383

[B15] Catanese J, Jaeger D (2021) Premotor ramping of thalamic neuronal activity is modulated by nigral inputs and contributes to control the timing of action release. J Neurosci 41:1878–1891. 10.1523/JNEUROSCI.1204-20.2020 33446518 PMC7939094

[B16] Chambers JM, Gurney K, Humphries M, Prescott A (2011) Mechanisms of choice in the primate brain: a quick look at positive feedback. In: *Modelling natural action selection* (Bryson JJ, Prescott TJ, Seth AK, eds), pp 390–420. Cambridge, UK: CUP.

[B17] Chevalier G, Deniau JM (1990) Disinhibition as a basic process in the expression of striatal function. Trends Neurosci 13:277–280. 10.1016/0166-2236(90)90109-N 1695403

[B18] Chevalier G, Vacher S, Deniau J, Desban M (1985) Disinhibition as a basic process in the expression of striatal function i. the striato-nigral influence on tecto-spinal/tecto-diencephalic neurons. Brain Res 334:215–226. 10.1016/0006-8993(85)90213-6 2859912

[B19] Churchland MM, Cunningham JP, Kaufman MT, Foster JD, Nuyujukian P, Ryu SI, Shenoy KV (2012) Neural population dynamics during reaching. Nature 487:51–56. 10.1038/nature11129 22722855 PMC3393826

[B20] Collins AGE, Frank MJ (2014) Opponent actor learning (opal): modeling interactive effects of striatal dopamine on reinforcement learning and choice incentive. Psychol Rev 121:337–366. 10.1037/a0037015 25090423

[B21] de A Marcelino AL, Gray O, Al-Fatly B, Gilmour W, Steele JD, Kühn AA, Gilbertson T (2023) Pallidal neuromodulation of the explore/exploit trade-off in decision-making. Elife 12:e79642. 10.7554/eLife.79642 36727860 PMC9940911

[B22] Deniau JM, Chevalier G (1985) Disinhibition as a basic process in the expression of striatal functions. ii. the striato-nigral influence on thalamocortical cells of the ventromedial thalamic nucleus. Brain Res 334:227–233. 10.1016/0006-8993(85)90214-8 3995318

[B23] Deniau JM, Chevalier G (1992) The lamellar organization of the rat substantia nigra pars reticulata: distribution of projection neurons. Neuroscience 46:361–377. 10.1016/0306-4522(92)90058-A 1542412

[B24] Ding L, Gold JI (2013) The basal ganglia’s contributions to perceptual decision making. Neuron 79:640–649. 10.1016/j.neuron.2013.07.042 23972593 PMC3771079

[B25] Dominey PF, Arbib MA (1992) A cortico-subcortical model for generation of spatially accurate sequential saccades. Cereb Cortex 2:153–175. 10.1093/cercor/2.2.153 1633413

[B26] Dominey P, Arbib M, Joseph J-P (1995) A model of corticostriatal plasticity for learning oculomotor associations and sequences. J Cogn Neurosci 1995:311–336. 10.1162/jocn.1995.7.3.311 23961864

[B27] Druckmann S, Chklovskii DB (2012) Neuronal circuits underlying persistent representations despite time varying activity. Curr Biol 22:2095–2103. 10.1016/j.cub.2012.08.058 23084992 PMC3543774

[B28] Dunovan K, Vich C, Clapp M, Verstynen T, Rubin J (2019) Reward-driven changes in striatal pathway competition shape evidence evaluation in decision-making. PLoS Comput Biol 15:e1006998. 10.1371/journal.pcbi.1006998 31060045 PMC6534331

[B29] Elber-Dorozko L, Loewenstein Y (2018) Striatal action-value neurons reconsidered. Elife 7:e34248. 10.7554/eLife.34248 29848442 PMC6008056

[B30] Fan D, Rossi MA, Yin HH (2012) Mechanisms of action selection and timing in substantia nigra neurons. J Neurosci 32:5534–5548. 10.1523/JNEUROSCI.5924-11.2012 22514315 PMC6703499

[B31] Felsen G, Mainen ZF (2008) Neural substrates of sensory-guided locomotor decisions in the rat superior colliculus. Neuron 60:137–148. 10.1016/j.neuron.2008.09.019 18940594 PMC2612727

[B32] Foster NN, et al. (2021) The mouse cortico-basal ganglia-thalamic network. Nature 598:188–194. 10.1038/s41586-021-03993-3 34616074 PMC8494639

[B33] Frank MJ (2005) Dynamic dopamine modulation in the basal ganglia: a neurocomputational account of cognitive deficits in medicated and nonmedicated parkinsonism. J Cogn Neurosci 17:51–72. 10.1162/0898929052880093 15701239

[B34] Friedlander T, Mayo AE, Tlusty T, Alon U (2015) Evolution of bow-tie architectures in biology. PLoS Comput Biol 11:e1004055. 10.1371/journal.pcbi.1004055 25798588 PMC4370773

[B35] Gallego JA, Perich MG, Miller LE, Solla SA (2017) Neural manifolds for the control of movement. Neuron 94:978–984. 10.1016/j.neuron.2017.05.025 28595054 PMC6122849

[B36] Girard B, Berthoz A (2005) From brainstem to cortex: computational models of saccade generation circuitry. Prog Neurobiol 77:215–251. 10.1016/j.pneurobio.2005.11.001 16343730

[B37] Goldberg JH, Farries MA, Fee MS (2013) Basal ganglia output to the thalamus: still a paradox. Trends Neurosci 36:695–705. 10.1016/j.tins.2013.09.001 24188636 PMC3855885

[B38] Gouvêa TS, Monteiro T, Motiwala A, Soares S, Machens C, Paton JJ (2015) Striatal dynamics explain duration judgments. Elife 4:e11386. 10.7554/eLife.11386 26641377 PMC4721960

[B39] Gu S, et al. (2015) Controllability of structural brain networks. Nat Commun 6:8414. 10.1038/ncomms9414 26423222 PMC4600713

[B40] Gulley JM, Kosobud AE, Rebec GV (2002) Behavior-related modulation of substantia nigra pars reticulata neurons in rats performing a conditioned reinforcement task. Neuroscience 111:337–349. 10.1016/S0306-4522(02)00018-0 11983319

[B41] Gulley JM, Kuwajima M, Mayhill E, Rebec GV (1999) Behavior-related changes in the activity of substantia nigra pars reticulata neurons in freely moving rats. Brain Res 845:68–76. 10.1016/S0006-8993(99)01932-0 10529445

[B42] Gurney KN, Humphries MD, Redgrave P (2015) A new framework for cortico-striatal plasticity: behavioural theory meets in vitro data at the reinforcement-action interface. PLoS Biol 13:e1002034. 10.1371/journal.pbio.1002034 25562526 PMC4285402

[B43] Gurney K, Prescott TJ, Redgrave P (2001) A computational model of action selection in the basal ganglia I: a new functional anatomy. Biol Cybern 85:401–410. 10.1007/PL00007984 11417052

[B44] Handel A, Glimcher PW (1999) Quantitative analysis of substantia nigra pars reticulata activity during a visually guided saccade task. J Neurophysiol 82:3458–3475. 10.1152/jn.1999.82.6.3458 10601475

[B45] Handel A, Glimcher PW (2000) Contextual modulation of substantia nigra pars reticulata neurons. J Neurophysiol 83:3042–3048. 10.1152/jn.2000.83.5.3042 10805699

[B46] Hart AS, Rutledge RB, Glimcher PW, Phillips PEM (2014) Phasic dopamine release in the rat nucleus accumbens symmetrically encodes a reward prediction error term. J Neurosci 34:698–704. 10.1523/JNEUROSCI.2489-13.2014 24431428 PMC3891951

[B47] Harvey CD, Coen P, Tank DW (2012) Choice-specific sequences in parietal cortex during a virtual-navigation decision task. Nature 484:62–68. 10.1038/nature10918 22419153 PMC3321074

[B48] Hennequin G, Ahmadian Y, Rubin DB, Lengyel M, Miller KD (2018) The dynamical regime of sensory cortex: stable dynamics around a single stimulus-tuned attractor account for patterns of noise variability. Neuron 98:846–860.e5. 10.1016/j.neuron.2018.04.017 29772203 PMC5971207

[B49] Hennequin G, Vogels TP, Gerstner W (2014) Optimal control of transient dynamics in balanced networks supports generation of complex movements. Neuron 82:1394–1406. 10.1016/j.neuron.2014.04.045 24945778 PMC6364799

[B50] Hikosaka O, Takikawa Y, Kawagoe R (2000) Role of the basal ganglia in the control of purposive saccadic eye movements. Physiol Rev 80:953–978. 10.1152/physrev.2000.80.3.953 10893428

[B51] Hikosaka O, Wurtz RH (1983a) Visual and oculomotor functions of monkey substantia nigra pars reticulata. I. relation of visual and auditory responses to saccades. J Neurophysiol 49:1230–1253. 10.1152/jn.1983.49.5.1230 6864248

[B52] Hikosaka O, Wurtz RH (1983b) Visual and oculomotor functions of monkey substantia nigra pars reticulata. III. memory-contingent visual and saccade responses. J Neurophysiol 49:1268–1284. 10.1152/jn.1983.49.5.1268 6864250

[B53] Hikosaka O, Wurtz RH (1983c) Visual and oculomotor functions of monkey substantia nigra pars reticulata. IV. relation of substantia nigra to superior colliculus. J Neurophysiol 49:1285–1301. 10.1152/jn.1983.49.5.1285 6306173

[B54] Hikosaka O, Wurtz RH (1985) Modification of saccadic eye movements by GABA-related substances. II. effects of muscimol in monkey substantia nigra pars reticulata. J Neurophysiol 53:292–308. 10.1152/jn.1985.53.1.292 2983038

[B55] Hoover JE, Strick PL (1993) Multiple output channels in the basal ganglia. Science 259:819–821. 10.1126/science.7679223 7679223

[B56] Houk J, Adams J, Barto A (1995) A model of how the basal ganglia generates and uses neural signals that predict reinforcement. In: *Models of information processing in the basal ganglia* (Houk J, Davis J, Beiser D, eds), pp 249–270. Cambridge, MA: MIT Press.

[B57] Howe MW, Dombeck DA (2016) Rapid signalling in distinct dopaminergic axons during locomotion and reward. Nature 535:505–510. 10.1038/nature18942 27398617 PMC4970879

[B58] Humphries MD, Gurney KN (2002) The role of intra-thalamic and thalamocortical circuits in action selection. Netw Comput Neural Syst 13:131–156. 10.1080/net.13.1.131.156 11873842

[B59] Humphries MD, Khamassi M, Gurney K (2012) Dopaminergic control of the exploration-exploitation trade-off via the basal ganglia. Front Neurosci 6:9. In “Decision Neuroscience” category - whatever Frontiers are calling their “journals” now…. 10.3389/fnins.2012.00009 22347155 PMC3272648

[B60] Humphries MD, Stewart RD, Gurney KN (2006) A physiologically plausible model of action selection and oscillatory activity in the basal ganglia. J Neurosci 26:12921–12942. 10.1523/JNEUROSCI.3486-06.2006 17167083 PMC6674973

[B61] Inagaki HK, Chen S, Ridder MC, Sah P, Li N, Yang Z, Hasanbegovic H, Gao Z, Gerfen CR, Svoboda K (2022) A midbrain-thalamus-cortex circuit reorganizes cortical dynamics to initiate movement. Cell 185:1065–1081.e23. 10.1016/j.cell.2022.02.006 35245431 PMC8990337

[B62] Ito M, Doya K (2011) Multiple representations and algorithms for reinforcement learning in the cortico-basal ganglia circuit. Curr Opin Neurobiol 21:368–373. 10.1016/j.conb.2011.04.001 21531544

[B63] Jiang H, Stein BE, McHaffie JG (2003) Opposing basal ganglia processes shape midbrain visuomotor activity bilaterally. Nature 423:982–986. 10.1038/nature01698 12827201

[B64] Jin X, Costa RM (2010) Start/stop signals emerge in nigrostriatal circuits during sequence learning. Nature 466:457–462. 10.1038/nature09263 20651684 PMC3477867

[B65] Joel D, Niv Y, Ruppin E (2002) Actor-critic models of the basal ganglia: new anatomical and computational perspectives. Neural Netw 15:535–547. 10.1016/S0893-6080(02)00047-3 12371510

[B66] Kao T-C, Hennequin G (2019) Neuroscience out of control: control-theoretic perspectives on neural circuit dynamics. Curr Opin Neurobiol 58:122–129. 10.1016/j.conb.2019.09.001 31563084

[B67] Kha HT, Finkelstein DI, Tomas D, Drago J, Pow DV, Horne MK (2001) Projections from the substantia nigra pars reticulata to the motor thalamus of the rat: single axon reconstructions and immunohistochemical study. J Comp Neurol 440:20–30. 10.1002/cne.1367 11745605

[B68] Khamassi M, Lacheze L, Girard B, Berthoz A, Guillot A (2005) Actor-critic models of reinforcement learning in the basal ganglia: from natural to artificial rats. Adapt Behav 13:131–148. 10.1177/105971230501300205

[B69] Klaus A, Alves da Silva J, Costa RM (2019) What, if, and when to move: basal ganglia circuits and self-paced action initiation. Annu Rev Neurosci 42:459–483. 10.1146/annurev-neuro-072116-031033 31018098

[B70] Kuramoto E, Fujiyama F, Nakamura KC, Tanaka Y, Hioki H, Kaneko T (2011) Complementary distribution of glutamatergic cerebellar and GABAergic basal ganglia afferents to the rat motor thalamic nuclei. Eur J Neurosci 33:95–109. 10.1111/j.1460-9568.2010.07481.x 21073550

[B71] Laughlin SB (2001) Energy as a constraint on the coding and processing of sensory information. Curr Opin Neurobiol 11:475–480. 10.1016/S0959-4388(00)00237-3 11502395

[B72] Leblois A, Boraud T, Meissner W, Bergman H, Hansel D (2006) Competition between feedback loops underlies normal and pathological dynamics in the basal ganglia. J Neurosci 26:3567–3583. 10.1523/JNEUROSCI.5050-05.2006 16571765 PMC6673853

[B73] Lee C, Rohrer WH, Sparks DL (1988) Population coding of saccadic eye movements by neurons in the superior colliculus. Nature 332:357–360. 10.1038/332357a0 3352733

[B74] Lee J, Wang W, Sabatini BL (2020) Anatomically segregated basal ganglia pathways allow parallel behavioral modulation. Nat Neurosci 23:1388–1398. 10.1038/s41593-020-00712-5 32989293 PMC7606600

[B75] Liénard J, Girard B (2014) A biologically constrained model of the whole basal ganglia addressing the paradoxes of connections and selection. J Comput Neurosci 36:445. 10.1007/s10827-013-0476-2 24077957

[B76] Lindahl M, Hellgren Kotaleski J (2016) Untangling basal ganglia network dynamics and function: role of dopamine depletion and inhibition investigated in a spiking network model. eNeuro 3:ENEURO.0156–16.2016. 10.1523/ENEURO.0156-16.2016 28101525 PMC5228592

[B77] Lintz MJ, Felsen G (2016) Basal ganglia output reflects internally-specified movements. Elife 5:e13833. 10.7554/eLife.13833 27377356 PMC4970866

[B78] Liu Y-Y, Slotine J-J, Barabási A-L (2011) Controllability of complex networks. Nature 473:167–173. 10.1038/nature10011 21562557

[B79] Logiaco L, Abbott LF, Escola S (2021) Thalamic control of cortical dynamics in a model of flexible motor sequencing. Cell Rep 35:109090. 10.1016/j.celrep.2021.109090 34077721 PMC8449509

[B80] Mahamed S, Garrison TJ, Shires J, Basso MA (2014) Stimulation of the substantia nigra influences the specification of memory-guided saccades. J Neurophysiol 111:804–816. 10.1152/jn.00002.2013 24259551 PMC3921393

[B81] McElvain LE, Chen Y, Moore JD, Brigidi GS, Bloodgood BL, Lim BK, Costa RM, Kleinfeld D (2021) Specific populations of basal ganglia output neurons target distinct brain stem areas while collateralizing throughout the diencephalon. Neuron 109:1721–1738.e4. 10.1016/j.neuron.2021.03.017 33823137 PMC8169061

[B82] Mello GBM, Soares S, Paton JJ (2015) A scalable population code for time in the striatum. Curr Biol 25:1113–1122. 10.1016/j.cub.2015.02.036 25913405

[B83] Middleton FA, Strick PL (2002) Basal-ganglia ‘projections’ to the prefrontal cortex of the primate. Cereb Cortex 12:926–935. 10.1093/cercor/12.9.926 12183392

[B84] Mink JW (1996) The basal ganglia: focused selection and inhibition of competing motor programs. Prog Neurobiol 50:381–425. 10.1016/S0301-0082(96)00042-1 9004351

[B85] Moll FW, Kranz D, Asensio AC, Elmaleh M, Ackert-Smith LA, Long MA (2023) Thalamus drives vocal onsets in the zebra finch courtship song. Nature 616:132–136. 10.1038/s41586-023-05818-x 36949189 PMC11967199

[B86] Möller M, Bogacz R (2019) Learning the payoffs and costs of actions. PLoS Comput Biol 15:e1006285. 10.1371/journal.pcbi.1006285 30818357 PMC6413954

[B87] Monteiro T, Rodrigues FS, Pexirra M, Cruz BF, Gonçalves AI, Rueda-Orozco PE, Paton JJ (2023) Using temperature to analyze the neural basis of a time-based decision. Nat Neurosci 26:1407–1416. 10.1038/s41593-023-01378-5 37443279

[B88] Morrissette AE, Chen P -H, Bhamani C, Borden PY, Waiblinger C, Stanley GB, Jaeger D (2019) Unilateral optogenetic inhibition and excitation of basal ganglia output affect directional lick choices and movement initiation in mice. Neuroscience 423:55–65. 10.1016/j.neuroscience.2019.10.031 31705892 PMC6917693

[B89] Nevet A, Morris G, Saban G, Arkadir D, Bergman H (2007) Lack of spike-count and spike-time correlations in the substantia nigra reticulata despite overlap of neural responses. J Neurophysiol 98:2232–2243. 10.1152/jn.00190.2007 17699698

[B90] Nishimura Y, Takada M, Mizuno N (1997) Topographic distribution and collateral projections of the two major populations of nigrothalamic neurons. a retrograde labeling study in the rat. Neurosci Res 28:1–9. 10.1016/S0168-0102(97)01171-1 9179875

[B91] Oh SW, et al. (2014) A mesoscale connectome of the mouse brain. Nature 508:207–214. 10.1038/nature13186 24695228 PMC5102064

[B92] Ölveczky BP, Andalman AS, Fee MS (2005) Vocal experimentation in the juvenile songbird requires a basal ganglia circuit. PLoS Biol 3:e153. 10.1371/journal.pbio.0030153 15826219 PMC1069649

[B93] Oorschot DE (1996) Total number of neurons in the neostriatal, pallidal, subthalamic, and substantia nigral nuclei of the rat basal ganglia: a stereological study using the cavalieri and optical disector methods. J Comp Neurol 366:580–99. 10.1002/(SICI)1096-9861(19960318)366:4<580::AID-CNE3>3.0.CO;2-0 8833111

[B94] Park J, Coddington LT, Dudman JT (2020) Basal ganglia circuits for action specification. Annu Rev Neurosci 43:485–507. 10.1146/annurev-neuro-070918-050452 32303147

[B95] Peters A, Payne BR (1993) Numerical relationships between geniculocortical afferents and pyramidal cell modules in cat primary visual cortex. Cereb Cortex 3:69–78. 10.1093/cercor/3.1.69 8439740

[B96] Rajan K, Abbott LF (2006) Eigenvalue spectra of random matrices for neural networks. Phys Rev Lett 97:188104. 10.1103/PhysRevLett.97.188104 17155583

[B97] Redgrave P, Prescott TJ, Gurney K (1999) The basal ganglia: a vertebrate solution to the selection problem? Neuroscience 89:1009–1023. 10.1016/S0306-4522(98)00319-4 10362291

[B98] Reynolds JN, Hyland BI, Wickens JR (2001) A cellular mechanism of reward-related learning. Nature 413:67–70. 10.1038/35092560 11544526

[B99] Rodarie D, Verasztó C, Roussel Y, Reimann M, Keller D, Ramaswamy S, Markram H, Gewaltig M-O (2022) A method to estimate the cellular composition of the mouse brain from heterogeneous datasets. PLoS Comput Biol 18:e1010739. 10.1371/journal.pcbi.1010739 36542673 PMC9838873

[B100] Rodriguez AC, Perich MG, Miller L, Humphries MD (2024) “Motor cortex latent dynamics encode spatial and temporal arm movement parameters independently.” *bioRxiv*.

[B101] Rossi MA, et al. (2016) A GABAergic nigrotectal pathway for coordination of drinking behavior. Nat Neurosci 19:742–748. 10.1038/nn.4285 27043290 PMC5014542

[B102] Rubin DB, Van Hooser SD, Miller KD (2015) The stabilized supralinear network: a unifying circuit motif underlying multi-input integration in sensory cortex. Neuron 85:402–417. 10.1016/j.neuron.2014.12.026 25611511 PMC4344127

[B103] Rudelson M, Vershynin R (2009) Smallest singular value of a random rectangular matrix. Commun Pure Appl Math 62:1707–1739. 10.1002/cpa.20294

[B104] Rueda-Orozco PE, Robbe D (2015) The striatum multiplexes contextual and kinematic information to constrain motor habits execution. Nat Neurosci 18:453–460. 10.1038/nn.3924 25622144 PMC4342106

[B105] Rutledge RB, Dean M, Caplin A, Glimcher PW (2010) Testing the reward prediction error hypothesis with an axiomatic model. J Neurosci 30:13525–13536. 10.1523/JNEUROSCI.1747-10.2010 20926678 PMC2957369

[B106] Sales-Carbonell C, Taouali W, Khalki L, Pasquet MO, Petit LF, Moreau T, Rueda-Orozco PE, Robbe D (2018) No discrete start/stop signals in the dorsal striatum of mice performing a learned action. Curr Biol 28:3044–3055.e5. 10.1016/j.cub.2018.07.038 30270180 PMC6231539

[B107] Samejima K, Ueda Y, Doya K, Kimura M (2005) Representation of action-specific reward values in the striatum. Science 310:1337–1340. 10.1126/science.1115270 16311337

[B108] Sato M, Hikosaka O (2002) Role of primate substantia nigra pars reticulata in reward-oriented saccadic eye movement. J Neurosci 22:2363–2373. 10.1523/JNEUROSCI.22-06-02363.2002 11896175 PMC6758246

[B109] Sauerbrei BA, Guo J -Z, Cohen JD, Mischiati M, Guo W, Kabra M, Verma N, Mensh B, Branson K, Hantman AW (2020) Cortical pattern generation during dexterous movement is input-driven. Nature 577:386–391. 10.1038/s41586-019-1869-9 31875851 PMC6962553

[B110] Schultz W, Dayan P, Montague PR (1997) A neural substrate of prediction and reward. Science 275:1593–1599. 10.1126/science.275.5306.1593 9054347

[B111] Schwab BC, Kase D, Zimnik A, Rosenbaum R, Codianni MG, Rubin JE, Turner RS (2020) Neural activity during a simple reaching task in macaques is counter to gating and rebound in basal ganglia-thalamic communication. PLoS Biol 18:e3000829. 10.1371/journal.pbio.3000829 33048920 PMC7584254

[B112] Sengupta B, Stemmler M, Laughlin SB, Niven JE (2010) Action potential energy efficiency varies among neuron types in vertebrates and invertebrates. PLoS Comput Biol 6:e1000840. 10.1371/journal.pcbi.1000840 20617202 PMC2895638

[B113] Shen W, Flajolet M, Greengard P, Surmeier DJ (2008) Dichotomous dopaminergic control of striatal synaptic plasticity. Science 321:848–851. 10.1126/science.1160575 18687967 PMC2833421

[B114] Sheth SA, Abuelem T, Gale JT, Eskandar EN (2011) Basal ganglia neurons dynamically facilitate exploration during associative learning. J Neurosci 31:4878–4885. 10.1523/JNEUROSCI.3658-10.2011 21451026 PMC3486636

[B115] Singh Alvarado J, Goffinet J, Michael V, Liberti W, Hatfield J, Gardner T, Pearson J, Mooney R (2021) Neural dynamics underlying birdsong practice and performance. Nature 599:635–639. 10.1038/s41586-021-04004-1 34671166 PMC9118926

[B116] Sontag E, Sussmann H (1997) Complete controllability of continuous-time recurrent neural networks. Syst Control Lett 30:177–183. 10.1016/s0167-6911(97)00002-9

[B117] Stocco A, Lebiere C, Anderson JR (2010) Conditional routing of information to the cortex: a model of the basal ganglia’s role in cognitive coordination. Psychol Rev 117:541–574. 10.1037/a0019077 20438237 PMC3064519

[B118] Sutton RS, Barto AG (2018) Reinforcement learning: an introduction, Ed 2. Cambridge, MA: MIT Press.

[B119] Thura D, Cabana J-F, Feghaly A, Cisek P (2022) Integrated neural dynamics of sensorimotor decisions and actions. PLoS Biol 20:e3001861. 10.1371/journal.pbio.3001861 36520685 PMC9754259

[B120] Thurat C, N’Guyen S, Girard B (2015) Biomimetic race model of the loop between the superior colliculus and the basal ganglia: subcortical selection of saccade targets. Neural Netw 67:54–73. 10.1016/j.neunet.2015.02.004 25884111

[B121] Tolhurst D, Movshon J, Dean A (1983) The statistical reliability of signals in single neurons in cat and monkey visual cortex. Vision Res 23:775–785. 10.1016/0042-6989(83)90200-6 6623937

[B122] Turner RS, Desmurget M (2010) Basal ganglia contributions to motor control: a vigorous tutor. Curr Opin Neurobiol 20:704–716. 10.1016/j.conb.2010.08.022 20850966 PMC3025075

[B123] Villalobos CA, Basso MA (2022) Optogenetic activation of the inhibitory nigro-collicular circuit evokes contralateral orienting movements in mice. Cell Rep 39:110699. 10.1016/j.celrep.2022.110699 35443172 PMC10144672

[B124] Vitay J, Hamker FH (2010) A computational model of basal ganglia and its role in memory retrieval in rewarded visual memory tasks. Front Comput Neurosci 4:13. 10.3389/fncom.2010.00013 20725505 PMC2901092

[B125] Voitov I, Mrsic-Flogel TD (2022) Cortical feedback loops bind distributed representations of working memory. Nature 608:381–389. 10.1038/s41586-022-05014-3 35896749 PMC9365695

[B126] Wärnberg E, Kumar A (2023) Feasibility of dopamine as a vector-valued feedback signal in the basal ganglia. Proc Natl Acad Sci U S A 120:e2221994120. 10.1073/pnas.2221994120 37527344 PMC10410740

[B127] Yartsev MM, Hanks TD, Yoon AM, Brody CD (2018) Causal contribution and dynamical encoding in the striatum during evidence accumulation. Elife 7:34929. 10.7554/eLife.34929 30141773 PMC6147735

[B128] Yttri EA, Dudman JT (2016) Opponent and bidirectional control of movement velocity in the basal ganglia. Nature 533:402–406. 10.1038/nature17639 27135927 PMC4873380

